# Control of Crystalline-Amorphous Structures of Polyhedral Oligomeric Silsesquioxanes Containing Two Types of Ammonium Side-Chain Groups and Their Properties as Protic Ionic Liquids

**DOI:** 10.3390/molecules24244553

**Published:** 2019-12-12

**Authors:** Ryoya Hasebe, Yoshiro Kaneko

**Affiliations:** Graduate School of Science and Engineering, Kagoshima University, 1-21-40 Korimoto, Kagoshima 890-0065, Japan; k7959128@kadai.jp

**Keywords:** silsesquioxane, POSS, ionic liquid

## Abstract

Polyhedral oligomeric silsesquioxanes (POSSs), **Am-POSS(x,y)**, prepared by hydrolytic condensation, contains two types of ammonium side-chain groups, where the numbering of x and y represents the type of ammonium ions in the POSS structure, corresponding to primary (**1**), secondary (**2**), tertiary (**3**), and quaternary (**4**) ammonium ions. Mixtures of the two starting materials selected from organotrialkoxysilanes containing primary, secondary, and tertiary amines and a quaternary ammonium salt [(RO)_3_Si(CH_2_)_3_R′, R = CH_3_ or CH_2_CH_3_, R′ = NH_2_, NHCH_3_, N(CH_3_)_2_, and N(CH_3_)_3_Cl] were dissolved in dimethyl sulfoxide (DMSO). The hydrolytic condensation was performed in the presence of bis(trifluoromethansulfonyl)imide (HNTf_2_) and water. All **Am-POSS(x,y)** structures consisted of a cage-type octamer (T_8_-POSS), as confirmed by ^29^Si NMR spectrometry and matrix-assisted laser desorption ionization-time of flight mass spectrometry (MALDI-TOF MS). Differential scanning calorimetry (DSC) and X-ray diffraction (XRD) analyses indicated that **Am-POSS(1,3)**, **Am-POSS(1,4)**, and **Am-POSS(2,4)** had amorphous structures. These POSSs have two or three differences in the number of methyl groups between the two types of ammonium side-chains. Conversely, **Am-POSS(1,2)**, **Am-POSS(2,3)**, and **Am-POSS(3,4)** had crystalline structures. The difference in the number of methyl groups between the two types of ammonium side-chains in these POSSs is only one. Therefore, the crystalline-amorphous structure of **Am-POSS(x,y)** is controlled by the side-chain group combinations. Furthermore, **Am-POSS(1,3)**, **Am-POSS(1,4)**, and **Am-POSS(2,4)** are protic ionic liquids with relatively low flow temperatures.

## 1. Introduction

Polyhedral oligomeric silsesquioxanes (POSSs), which are generally prepared by the hydrolytic condensation of trifunctional silane compounds, such as organotrialkoxysilanes and organotrichlorosilanes, have attracted much attention in recent years as compounds having both inorganic and organic characteristics. In addition to the remarkable thermal and chemical stability derived from siloxane-bond frameworks, good dispersibility and solubility are provided due to regulated cage structures and suitable side-chain groups [1,2,3,4]. POSSs are, therefore, in great demand as dispersible inorganic compounds for the development of organic—inorganic hybrid materials [5,6,7,8,9,10].

Well-known POSSs are typically crystalline, cage-type octamers, because of their isotropic cubic structure. The amorphization of POSSs is one of the recent interesting topics in basic research and material applications. For example, amorphous POSS dimers [11] and polymers [12,13,14] are prepared by linking a plurality of POSSs, and are applied as transparent thermostable materials. There are some examples of large-sized POSSs (e.g., cage-type decamers) [15,16] and incomplete POSSs [17], which are also amorphous.

As a simple method to amorphize POSSs, we have prepared POSS with randomly arranged two types of ammonium side-chain groups (3-aminopropyl and 3-(2-aminoethylamino)propyl groups protonated with trifluoromethanesulfonic acid (HOTf)) [18]. In addition, it was also found that quaternary ammonium and imidazolium salt-containing POSS is amorphous, having no melting point (*T*_m_) [19]. This POSS had a glass transition point (*T*_g_) of −8 °C, and the temperature at which it tilted and flowed was 30 °C, showing the property of room temperature ionic liquid (IL).

As mentioned above, amorphous POSSs with randomly arranged two types of side-chain groups exhibited characteristics that are relevant for various material applications. However, no detailed investigation has been performed on how side-chain combinations affect the amorphous or crystalline morphology.

In this study, the effect of combinations of the two side-chains on the crystalline-amorphous structure of the POSSs containing two types of ammonium side-chain groups (**Am-POSS(x,y)**) was investigated, where the numbering of x and y represents the type of ammonium ions in the POSS structure, corresponding to primary (**1**), secondary (**2**), tertiary (**3**), and quaternary (**4**) ammonium ions. In addition, the properties of **Am-POSS(x,y)** as ionic liquids (ILs) were evaluated.

## 2. Results and Discussion

### 2.1. Preparation and Characterization of POSSs Containing One Type of Ammonium Side-Chain Group (**Am-POSS(x)**)

For comparison, prior to the preparation and characterization of **Am-POSS(x,y)**, POSSs containing one type of ammonium side-chain group (**Am-POSS(x)**, where the numbering of x represented the type of ammonium ion in the POSS structure, corresponding to primary (**1**), secondary (**2**), tertiary (**3**), and quaternary (**4**) ammonium ions) were prepared and characterized. In our previous studies on the preparation of ammonium-functionalized POSSs using superacid catalysts, the solvent water was yielded a mixture of cage-type octamer (main product) and decamer (minor product) [20], while applying the solvent dimethyl sulfoxide (DMSO) selectively provided only a cage-type octamer in higher yield at short reaction times [21]. Therefore, DMSO was used as a solvent in this study.

**Scheme 1:**
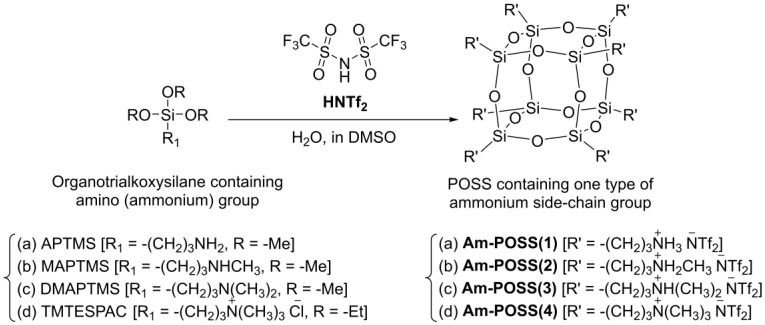
Preparation of POSSs containing one type of ammonium side-chain group: (**a**) **Am-POSS(1)**, (**b**) **Am-POSS(2)**, (**c**) **Am-POSS(3)**, and (**d**) **Am-POSS(4)**.

**Am-POSS(x)**s were prepared in DMSO by the hydrolytic condensation of 3-aminopropyltrimethoxysilane (APTMS), 3-(methylamino)propyltrimethoxysilane (MAPTMS), 3-(dimethylamino)propyltrimethoxysilane (DMAPTMS), and trimethyl [3-(triethoxysilyl)propyl]ammonium chloride (TMTESPAC), respectively, in the presence of bis(trifluoromethansulfonyl)imide (HNTf_2_) and water (the feed molar ratio of amine:HNTf_2_:H_2_O was 1.0:1.5:5.0) (Scheme 1). Solubilities of **Am-POSS(x)** are summarized in Table 1. All POSSs were soluble in highly polar organic solvents but insoluble in low polarity organic solvents. POSSs, except **Am-POSS(1)**, was also insoluble in water.

**Figure 1:**
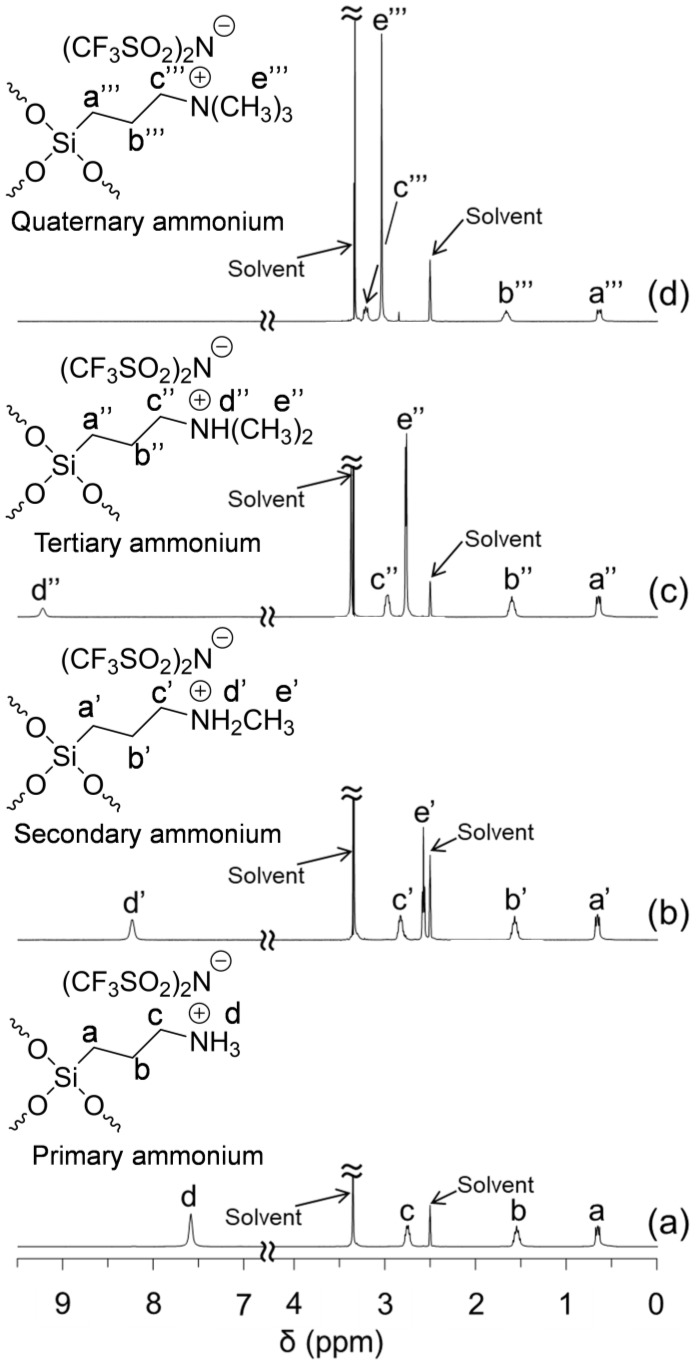
^1^H NMR spectra of (**a**) **Am-POSS(1)**, (**b**) **Am-POSS(2)**, (**c**) **Am-POSS(3)**, and (**d**) **Am-POSS(4)** in DMSO-*d*_6_. Chemical shifts were referenced to DMSO (δ 2.5).

The ^1^H NMR spectra of **Am-POSS(x)** in DMSO-*d*_6_ showed the signals attributable to the side-chains of the corresponding POSSs (Figure 1). The signals for the methoxy or ethoxy group of the starting materials were not observed (Figure 1), pointing out that the starting materials were not present in the products. The energy-dispersive X-ray (EDX) pattern of **Am-POSS(4)** contained no peaks originating from Cl (*ca*. 2.6 and 2.8 keV). The Si:S atom ratios were estimated to be 1.00:1.95 for **Am-POSS(1)**, 1.00:2.02 for **Am-POSS(2)**, 1.00:1.96 for **Am-POSS(3)**, and 1.00:2.00 for **Am-POSS(4)**, respectively, indicating that the molar ratios of ammonium cations to NTf_2_ anions in all products were ca. 1:1 (Figure S1).

**Figure 2:**
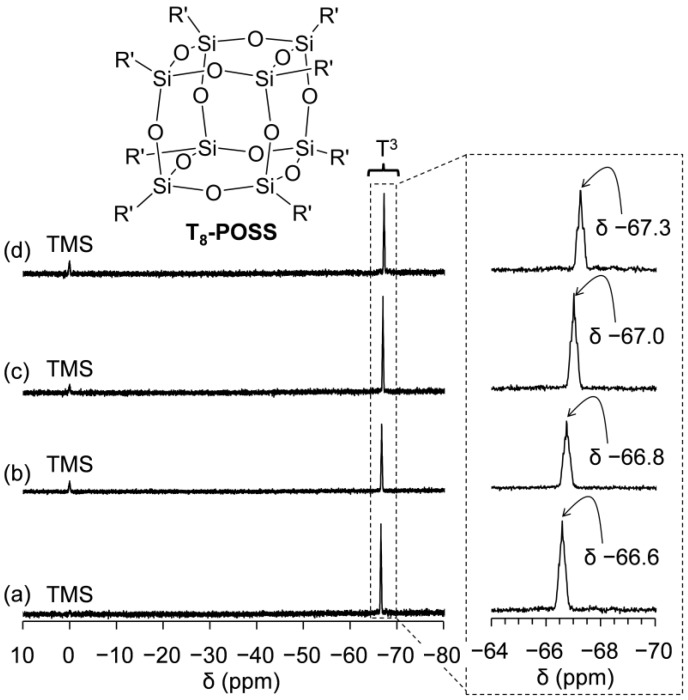
^29^Si NMR spectra of (**a**) **Am-POSS(1)**, (**b**) **Am-POSS(2)**, (**c**) **Am-POSS(3)**, and (**d**) **Am-POSS(4)** in DMSO-*d*_6_ at 40 °C. Chemical shifts were referenced to tetramethylsilane (TMS) (δ 0.0).

The ^29^Si NMR spectra of **Am-POSS(x)** in DMSO-*d*_6_ at 40 °C showed only the sharp signals due to the T^3^ structure (C*Si*(OSi)*_n_*(OH)_3–*n*_, *n* = 3) in the range between δ −66.6 and −67.3 ppm. These peaks are attributable to cage-type octamer (T_8_-POSS) (Figure 2). To support these ^29^Si NMR results, the matrix-assisted laser desorption ionization-time of flight mass spectrometry (MALDI-TOF MS) was performed on these POSSs. The MALDI-TOF MS of **Am-POSS(1)**, **Am-POSS(2)**, **Am-POSS(3)**, and **Am-POSS(4)** showed several peaks corresponding to the masses of T_8_-POSS (Figures S2–S5). These results indicated that the products consisted of T_8_-POSS only, no other POSSs, such as cage-type decamer (T_10_-POSS) and cage-type dodecamer (T_12_-POSS), were present in the products.

### 2.2. Evaluation of Crystalline-Amorphous Structures of **Am-POSS(x)**

**Figure 3:**
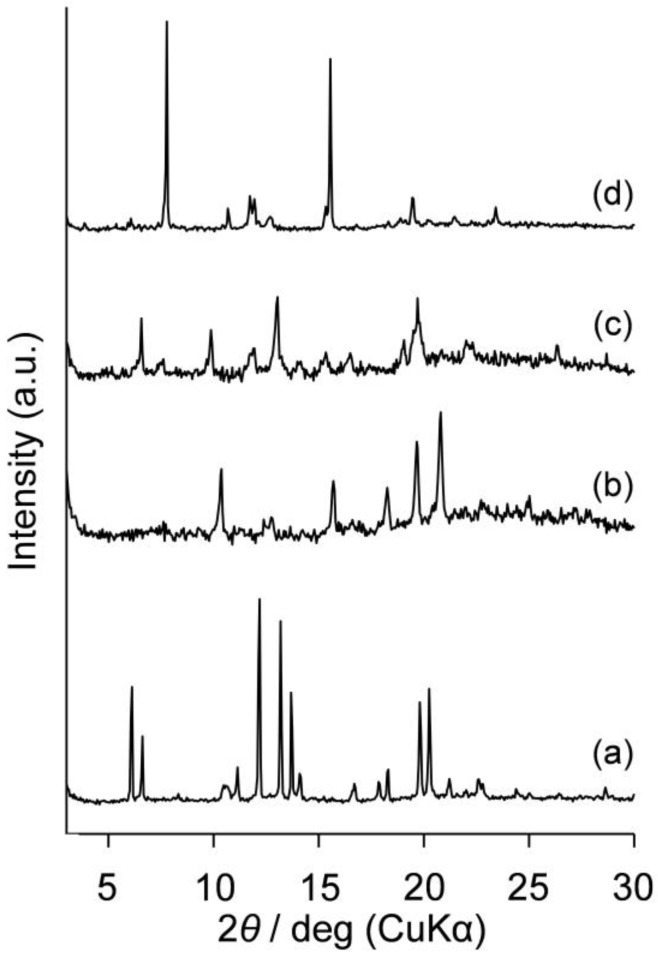
XRD patterns (immediately after preparation) obtained at room temperature (*ca*. 25 °C) of (**a**) **Am-POSS(1)**, (**b**) **Am-POSS(2)**, (**c**) **Am-POSS(3)**, and (**d**) **Am-POSS(4).** The amount of each product on the glass was ca. 1.0 mg·cm^−2^.

**Figure 4:**
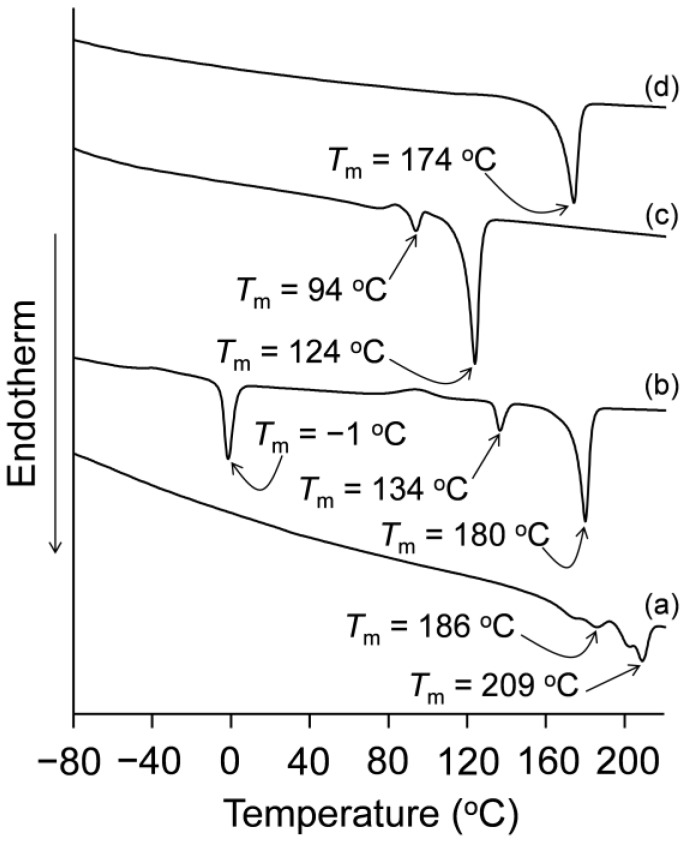
DSC curves of (**a**) **Am-POSS(1)**, (**b**) **Am-POSS(2)**, (**c**) **Am-POSS(3)**, and (**d**) **Am-POSS(4)**.

The X-ray diffraction (XRD) patterns of all **Am-POSS(x)** showed sharp diffraction peaks, indicating their crystalline structures (Figure 3). In addition, in the differential scanning calorimetry (DSC) curves of **Am-POSS(1)**, **Am-POSS(2)**, **Am-POSS(3)**, and **Am-POSS(4)**, the endothermic peaks due to *T*_m_s were observed at 186 and 209 °C (Figure 4a), −1, 134, and 180 °C (Figure 4b), 94 and 124 °C (Figure 4c), and 174 °C (Figure 4d), respectively. Multiple endothermic peaks were observed for POSSs, except **Am-POSS(4)**, potentially caused by the different crystalline forms of these POSSs. A more detailed study on the crystalline forms of these POSSs containing one type of ammonium side-chain group is currently in progress.

### 2.3. Preparation and Characterization of POSSs Containing Two Types of Ammonium Side-Chain Groups (**Am-POSS(x,y)**)

**Scheme 2:**
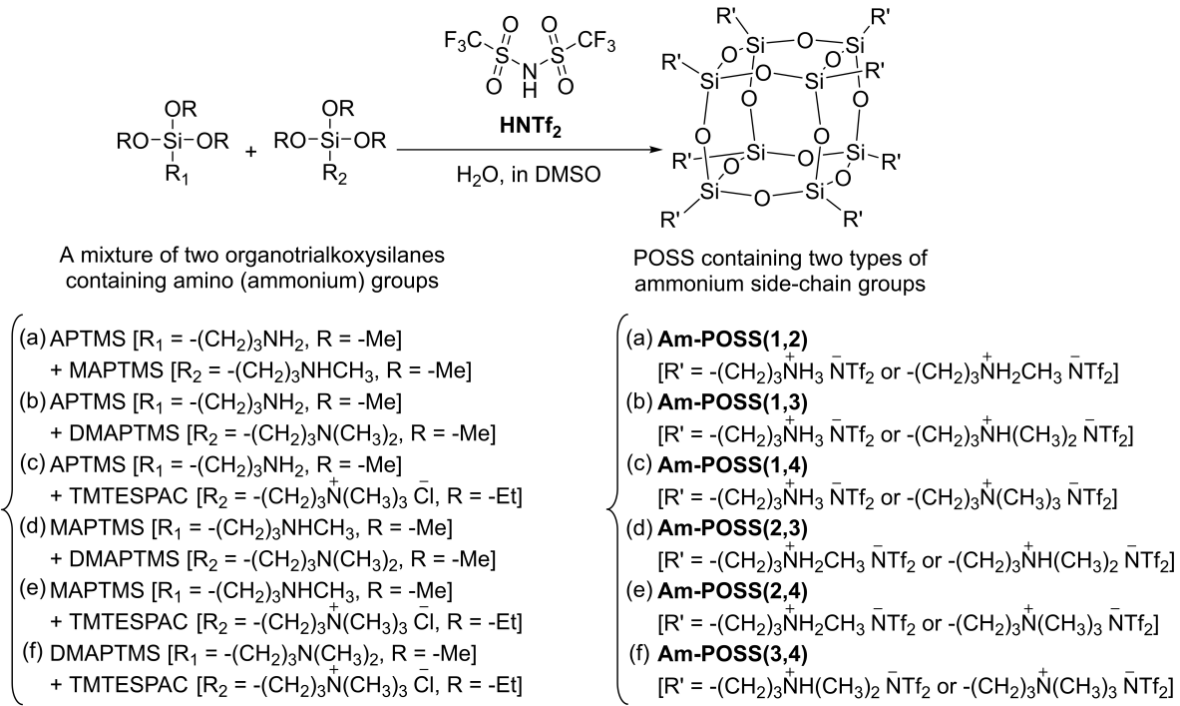
Preparation of POSSs containing two types of ammonium side-chain groups: (**a**) **Am-POSS(1,2)**, (**b**) **Am-POSS(1,3)**, (**c**) **Am-POSS(1,4)**, (**d**) **Am-POSS(2,3)**, (**e**) **Am-POSS(2,4)**, and (**f**) **Am-POSS(3,4)**.

**Am-POSS(x,y)**s were prepared by the hydrolytic condensation of a mixture of two starting materials selected from APTMS, MAPTMS, DMAPTMS, and TMTESPAC. DMSO was used as a solvent together with HNTf_2_ and water (the feed molar ratio of amine: HNTf_2_:H_2_O was 1.0:1.5:5.0) (Scheme 2). Solubilities of **Am-POSS(x,y)** are summarized in Table 2. All **Am-POSS(x,y)** were soluble in highly polar organic solvents but insoluble in water and low polarity organic solvents.

**Figure 5:**
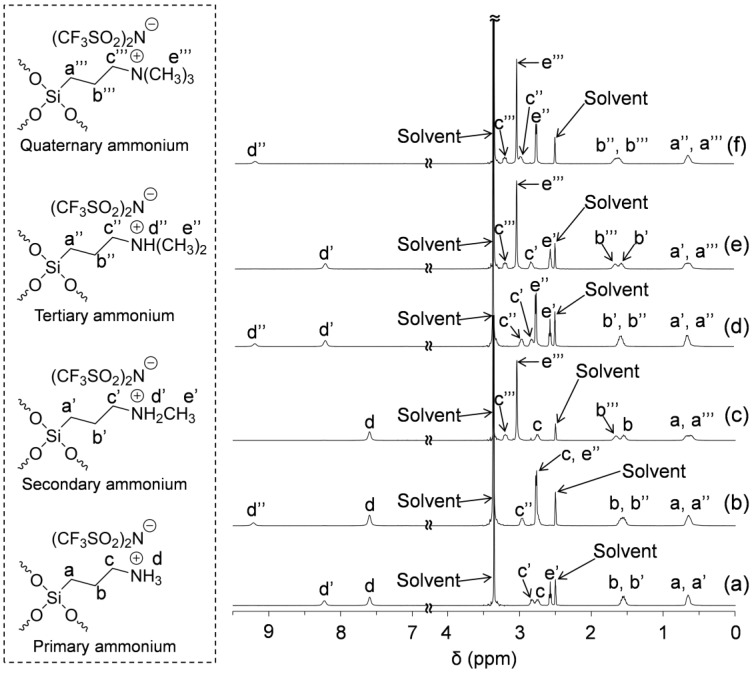
^1^H NMR spectra of (**a**) **Am-POSS(1,2)**, (**b**) **Am-POSS(1,3)**, (**c**) **Am-POSS(1,4)**, (**d**) **Am-POSS(2,3)**, (**e**) **Am-POSS(2,4)**, and (**f**) **Am-POSS(3,4)** in DMSO-*d*_6_. Chemical shifts were referenced to DMSO (δ 2.5).

The ^1^H NMR spectra of **Am-POSS(x,y)** in DMSO-*d*_6_ showed the signals attributable to the side-chains of both the two selected organotrialkoxysilane components (Figure 5). The signals for the methoxy or ethoxy group of the two starting materials were not observed (Figure 5). The compositional ratio of the two starting material components in **Am-POSS(x,y)** was estimated to be ca. 1:1, considering the integrated ratio of the signals in the ^1^H NMR spectrum, i.e., (a + a′):e′ (4.00:2.96) for **Am-POSS(1,2)**, (a + a″):c″ (4.00:2.01) for **Am-POSS(1,3)**, c:c‴ (2.00:2.07) for **Am-POSS(1,4)**, (a′ + a″):c″ (4.00:1.99) for **Am-POSS(2,3)**, c′:c‴ (2.00:2.00) for **Am-POSS(2,4)**, and (a″ + a‴):c‴ (4.00:2.02) for **Am-POSS(3,4)**. The EDX patterns of **Am-POSS(1,4)**, **Am-POSS(2,4)**, and **Am-POSS(3,4)** contained no peaks originating from Cl (*ca*. 2.6 and 2.8 keV). The Si:S atom ratios were estimated to be 1.00:1.98 for **Am-POSS(1,2)**, 1.00:2.03 for **Am-POSS(1,3)**, 1.00:2.00 for **Am-POSS(1,4)**, 1.00:2.06 for **Am-POSS(2,3)**, 1.00:2.00 for **Am-POSS(2,4)**, and 1.00:1.98 for **Am-POSS(3,4)**, respectively, indicating that the molar ratios of ammonium cations to NTf_2_ anions in all products were ca. 1:1 (Figure S6).

**Figure 6:**
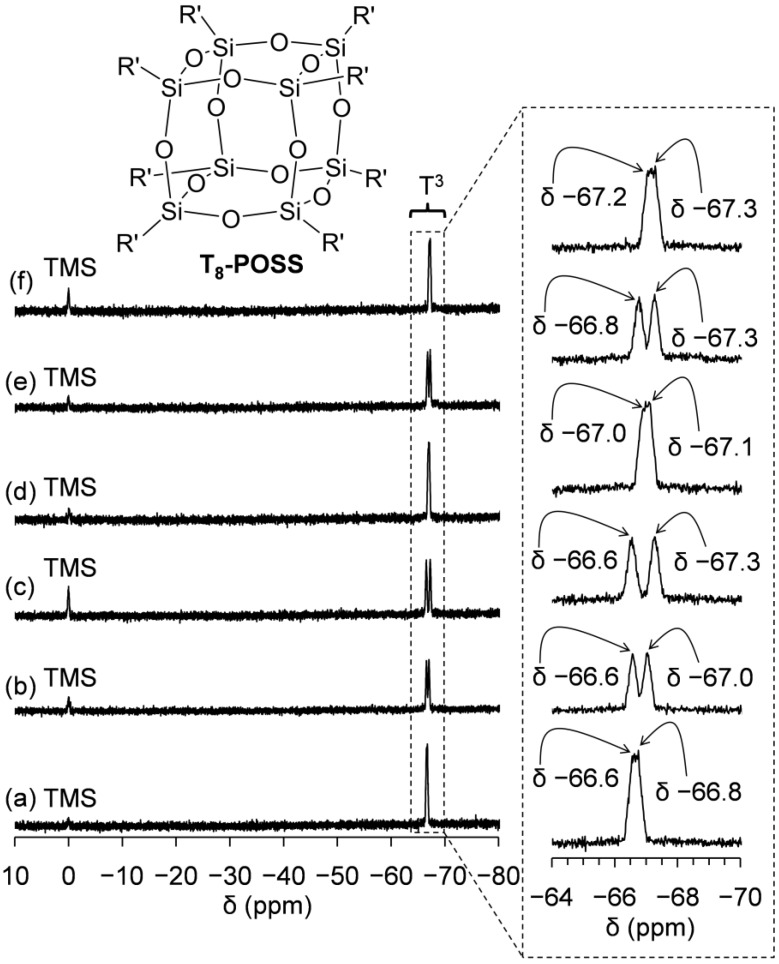
^29^Si NMR spectra of (**a**) **Am-POSS(1,2)**, (**b**) **Am-POSS(1,3)**, (**c**) **Am-POSS(1,4)**, (**d**) **Am-POSS(2,3)**, (**e**) **Am-POSS(2,4)**, and (**f**) **Am-POSS(3,4)** in DMSO-*d*_6_ at 40 °C. Chemical shifts were referenced to tetramethylsilane (TMS) (δ 0.0).

The ^29^Si NMR spectra of **Am-POSS(x,y)** in DMSO-*d*_6_ at 40 °C showed only the sharp signals in the range between δ −66.6 and −67.3 ppm, corresponding to the T^3^ structure (Figure 6). These peaks correspond to cage-type octamer (T_8_-POSS). To support these ^29^Si NMR results, MALDI-TOF MS was performed on these POSSs. The MALDI-TOF MS of all **Am-POSS(x,y)** showed several peaks ascribed to the masses of T_8_-POSS (Figures S7–S12). In most POSSs, the number of combinations of the two side-chain groups ranged from 1:7 to 7:1. This indicates that the products consisted of T_8_-POSS only, no other POSSs, such as T_10_- and T_12_-POSSs were present.

### 2.4. Evaluation of Crystalline-Amorphous Structures of **Am-POSS(x,y)**

**Figure 7:**
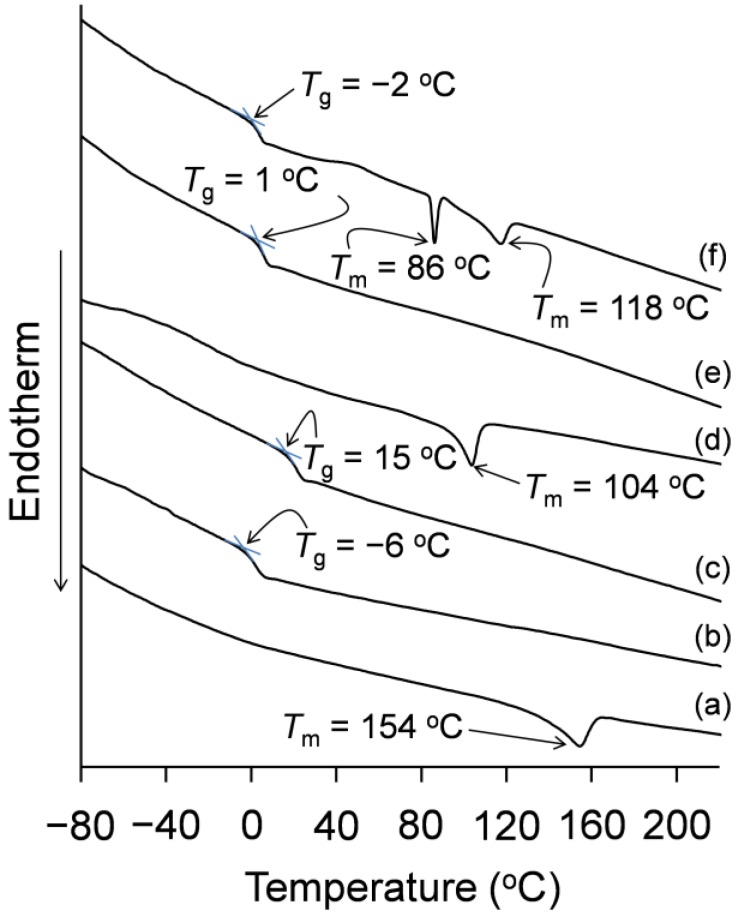
DSC curves of (**a**) **Am-POSS(1,2)**, (**b**) **Am-POSS(1,3)**, (**c**) **Am-POSS(1,4)**, (**d**) **Am-POSS(2,3)**, (**e**) **Am-POSS(2,4)**, and (**f**) **Am-POSS(3,4)**.

**Figure 8:**
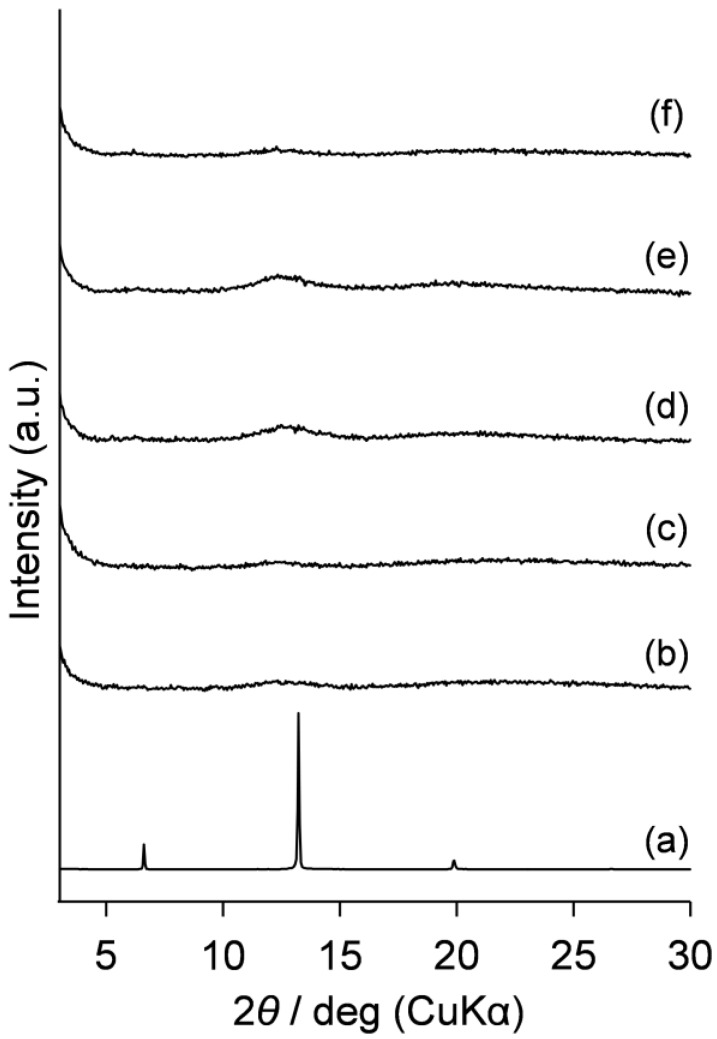
XRD patterns (immediately after preparation) obtained at room temperature (*ca*. 25 °C) of (**a**) **Am-POSS(1,2)**, (**b**) **Am-POSS(1,3)**, (**c**) **Am-POSS(1,4)**, (**d**) **Am-POSS(2,3)**, (**e**) **Am-POSS(2,4)**, and (**f**) **Am-POSS(3,4)**. The amount of each product on the glass was ca. 1.0 mg·cm^−2^.

**Figure 9:**
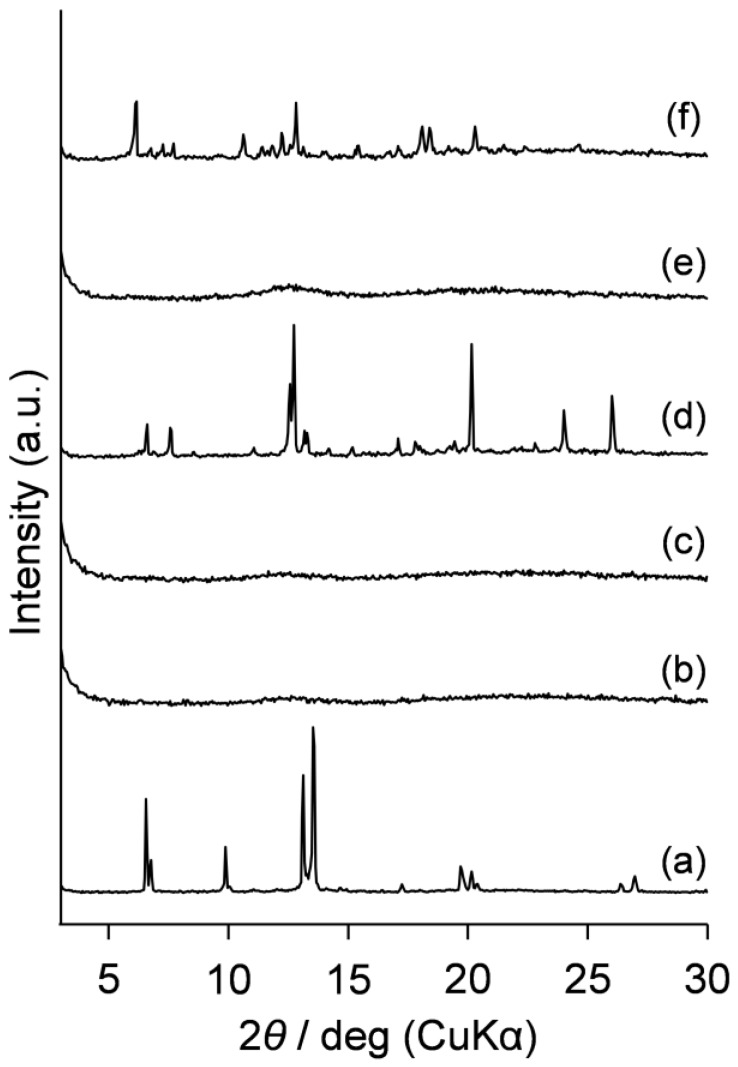
XRD patterns (standing for more than 2 weeks) obtained at room temperature (*ca*. 25 °C) of (**a**) **Am-POSS(1,2)**, (**b**) **Am-POSS(1,3)**, (**c**) **Am-POSS(1,4)**, (**d**) **Am-POSS(2,3)**, (**e**) **Am-POSS(2,4)**, and (**f**) **Am-POSS(3,4)**. The amount of each product on the glass was ca. 1.0 mg·cm^−2^.

In the DSC curves of **Am-POSS(1,3)**, **Am-POSS(1,4)**, and **Am-POSS(2,4)**, baseline shifts due to *T*_g_s were observed at −6 °C (Figure 7b, Run 2 in Table 3), 15 °C (Figure 7c, Run 3 in Table 3), and 1 °C (Figure 7e, Run 5 in Table 3), respectively, and endothermic peaks due to *T*_m_s were not observed, suggesting amorphous structures. Conversely, **Am-POSS(1,2)**, **Am-POSS(2,3)**, and **Am-POSS(3,4)** had *T*_m_s at 154 °C (Figure 7a, Run 1 in Table 3), 104 °C (Figure 7d, Run 4 in Table 3), and 86 and 118 °C (Figure 7f, Run 6 in Table 3), respectively, indicating crystalline structures. In the XRD patterns of these products, immediately recorded after preparation, sharp diffraction peaks were observed only for **Am-POSS(1,2)** (Figure 8a). Repeating the XRD after more than two weeks resulted in sharp diffraction peaks for **Am-POSS(2,3)** and **Am-POSS(3,4)** (Figure 9d,f). Although the crystallization rate was different, **Am-POSS(1,2)**, **Am-POSS(2,3)**, and **Am-POSS(3,4)** are crystalline compounds. On the other hand, **Am-POSS(1,3)**, **Am-POSS(1,4)**, and **Am-POSS(2,4)** did not show any diffraction peaks, indicating that these compounds have amorphous structures. These XRD results did support the DSC results.

Based on the DSC and XRD results, we concluded that crystalline and amorphous POSSs were selectively prepared, depending on the combination of side-chain groups. **Am-POSS(1,3)**, **Am-POSS(1,4)**, and **Am-POSS(2,4)** have two or three differences in the number of methyl groups between two types of ammonium side-chains. Because of the low molecular symmetry of the POSSs containing two different randomly distributed side-chain groups, crystallization was suppressed, leading to amorphous structures. Conversely, the molecular symmetry of **Am-POSS(1,2)**, **Am-POSS(2,3)**, and **Am-POSS(3,4)** were maintained because of the difference in the numbers of methyl groups between the two types of ammonium side-chains was only one. Therefore, it seems difficult to suppress the crystallization of these POSSs.

### 2.5. Evaluation of **Am-POSS(x,y)** as ILs

ILs are generally defined as salts that melt below 100 °C (there is also a definition of less than 150 °C). The fundamental thermodynamic properties of ILs have been extensively studied [22,23,24] for applications, such as green solvents [25,26,27,28] and electrolyte materials [29,30,31]. ILs present unique properties, such as high thermal stability, low vapor pressure, and high ionic conductivity. ILs are classified as aprotic ILs (AILs) or protic ILs (PILs), depending on the absence or presence of active protons. PILs are prepared by a simple neutralization (proton transfer) reaction between a Brønsted acid and a Brønsted base [32,33]. Upon heating, PILs formation can be easily reversed to obtain the original acid and base, showing inferior thermal stability to that of AILs. Therefore, the thermal stability of PILs needs to be improved.

A POSS with IL properties was first developed by Chujo, Tanaka, and co-workers [34]. This POSS contained anionic carboxylate side-chains and imidazolium cations as counterions, and its melting point (*T*_m_) was 23 °C. Another IL containing a POSS structure was reported by Feng, Zhang, and co-workers. It contained cationic imidazolium side-chains and dodecyl sulfate anions as counterions and had a *T*_m_ of 18 °C [35]. We have also reported the preparation of ILs containing randomly structured silsesquioxanes [36,37] and POSS with two types of side-chains [19]. However, all these ILs containing silsesquioxane frameworks were not PILs but AILs.

Although our group reported that cyclic oligosiloxanes containing 3-aminopropyl or 3-(2-aminoethylamino)propyl side-chain groups protonated with HNTf_2_ were of PILs nature [38], POSS containing the same side-chain groups did not show IL characteristics, because of the high crystallinity. The amorphous **Am-POSS(1,3)**, **Am-POSS(1,4)**, and **Am-POSS(2,4)** prepared in this study have active protons and showed PILs characteristics.

**Figure 10:**
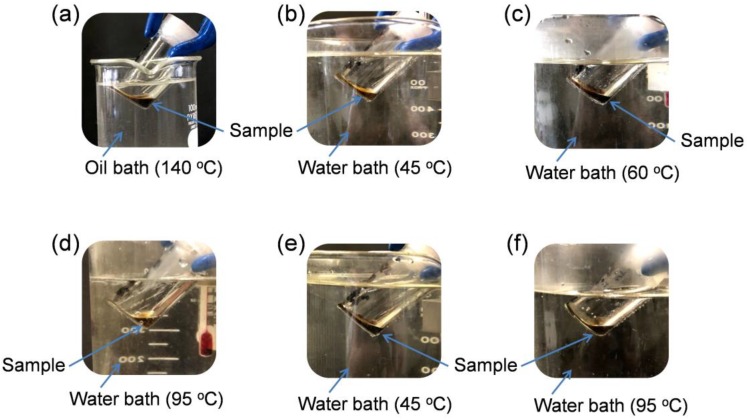
Photographs of flow temperature measurements using (**a**) **Am-POSS(1,2)**, (**b**) **Am-POSS(1,3)**, (**c**) **Am-POSS(1,4)**, (**d**) **Am-POSS(2,3)**, (**e**) **Am-POSS(2,4)**, and (**f**) **Am-POSS(3,4)**.

The flow temperatures of POSSs were confirmed as follows: Samples in glass vessels were maintained in a horizontal position at 200 °C for 15 min, and then cooled to room temperature (*ca*. 25 °C). Next, the vessels were kept horizontally at various temperatures (with 5 °C intervals) for 10 min and then tilted for 15 min. Following this procedure, it was confirmed that **Am-POSS(1,2)**, **Am-POSS(1,3)**, **Am-POSS(1,4)**, **Am-POSS(2,3)**, **Am-POSS(2,4)**, and **Am-POSS(3,4)** flowed at 140 °C (Figure 10a, Run 1 in Table 3), 45 °C (Figure 10b, Run 2 in Table 3), 60 °C (Figure 10c, Run 3 in Table 3), 95 °C (Figure 10d, Run 4 in Table 3), 45 °C (Figure 10e, Run 5 in Table 3), and 95 °C (Figure 10f, Run 6 in Table 3), respectively. These results indicated that amorphous **Am-POSS(1,3)**, **Am-POSS(1,4)**, and **Am-POSS(2,4)** were PILs with relatively low flow temperatures, while crystalline **Am-POSS(1,2)**, **Am-POSS(2,3)**, and **Am-POSS(3,4)** were PILs with relatively high flow temperatures.

Finally, the thermal stabilities of **Am-POSS(x,y)** were investigated by thermogravimetric analyses (TGA) (Figure S13). The pyrolysis temperatures of 5% (*T*_d5_) and 10% (*T*_d10_) weight losses of **Am-POSS(x,y)** are summarized in Table 3. It was found that the *T*_d5_ values of all **Am-POSS(x,y)** exceeded 370 °C. These values indicate excellent thermal stabilities and results from the suppression of the molecular tumbling by the silsesquioxane framework [34].

## 3. Materials and Methods

### 3.1. Materials

APTMS, MAPTMS, DMAPTMS, TMTESPAC, tris(2,4-pentanedionato)chromium(III) [Cr(acac)_3_], and tetramethylsilane (TMS) were purchased from Tokyo Chemical Industry Co., Ltd. (Tokyo, Japan). HNTf_2_ was purchased from Kanto Chemical Co., Inc. (Tokyo, Japan). 2,5-Dihydroxybenzoic acid (DHB) was purchased from Bruker Daltonik Gmbh (Bremen, Germany). Other reagents and solvents were purchased from FUJIFILM Wako Pure Chemical Co., Ltd. (Osaka, Japan). All reagents and solvents were used without further purification.

### 3.2. Preparation of **Am-POSS(1)**

A mixture of a 0.5 mol·L^−1^ DMSO solution of HNTf_2_ (6.0 mL, 3.0 mmol) and water (0.180 g, 10.0 mmol) was added to APTMS (purity: 96%, 0.374 g, 2.0 mmol) while stirring at room temperature (*ca*. 25 °C). The resulting solution was further stirred for 2 h. This solution was then heated at ca. 60 °C on a hot plate for 15 h using a disposal tray made of polypropylene (PP), as shown in Figure S14, and the resulting viscous liquid was maintained at 100 °C for 2 h. Subsequently, the crude product was dissolved in methanol (3 mL) at room temperature (*ca*. 25 °C), and this solution was poured into an acetone-chloroform mixed solvent (1:19 *v/v*, 180 mL). The insoluble part was isolated by decantation and washed with chloroform (*ca*. 50 mL, 5 times). To collect the material in a glass container, after dissolving it in methanol, the solution was transferred into a glass container and evaporated. The resulting product was then maintained at 200 °C for 5 h to yield 0.761 g of a white solid product (97% yield based on the ideal chemical formula of one unit of this product, i.e., [SiO_1.5_(CH_2_)_3_NH_3_ (CF_3_SO_2_)_2_N, FW = 391.4]). ^1^H NMR (400 MHz, DMSO-*d*_6_): δ 7.75–7.42 (3H, br, –SiCH_2_CH_2_CH_2_N*H*_3_), δ 2.81–2.65 (2H, br, –SiCH_2_CH_2_C*H*_2_NH_3_), δ 1.62–1.40 (2H, br, –SiCH_2_C*H*_2_CH_2_NH_3_), δ 0.65 (2H, t, J = 8.24 Hz, –SiC*H*_2_CH_2_CH_2_NH_3_). ^29^Si NMR (79.4 MHz, DMSO-*d*_6_): δ −66.6 (T^3^, T_8_-POSS).

### 3.3. Preparation of **Am-POSS(2)**

**Am-POSS(2)** was prepared similar to **Am-POSS(1)**, except that the starting material was MAPTMS (purity: 95%, 0.407 g, 2.0 mmol). A white solid product (0.679 g) was obtained (84% yield based on the ideal chemical formula of one unit of this product, i.e., [SiO_1.5_(CH_2_)_3_NH_2_CH_3_ (CF_3_SO_2_)_2_N, FW = 405.4]). ^1^H NMR (400 MHz, DMSO-*d*_6_): δ 8.35–8.09 (2H, br, –SiCH_2_CH_2_CH_2_N*H*_2_CH_3_), δ 2.89–2.75 (2H, br, –SiCH_2_CH_2_C*H*_2_NH_2_CH_3_), δ 2.57 (3H, t, J = 5.50 Hz, –SiCH_2_CH_2_CH_2_NH_2_C*H*_3_), δ 1.65–1.46 (2H, br, –SiCH_2_C*H*_2_CH_2_NH_2_CH_3_), δ 0.65 (2H, t, J = 8.24 Hz, –SiC*H*_2_CH_2_CH_2_NH_2_CH_3_). ^29^Si NMR (79.4 MHz, DMSO-*d*_6_): δ −66.8 (T^3^, T_8_-POSS).

### 3.4. Preparation of **Am-POSS(3)**

**Am-POSS(3)** was prepared similar to **Am-POSS(1)**, except that the starting material was DMAPTMS (purity: 96%, 0.432 g, 2.0 mmol). A white solid product (0.807 g) was obtained (96% yield based on the ideal chemical formula of one unit of this product, i.e., [SiO_1.5_(CH_2_)_3_NH(CH_3_)_2_ (CF_3_SO_2_)_2_N, FW = 419.4]). ^1^H NMR (400 MHz, DMSO-*d*_6_): δ 9.37–9.05 (1H, br, –SiCH_2_CH_2_CH_2_N*H*(CH_3_)_2_), δ 3.03–2.90 (2H, br, –SiCH_2_CH_2_C*H*_2_NH(CH_3_)_2_), δ 2.83–2.65 (6H, br, –SiCH_2_CH_2_CH_2_NH(C*H*_3_)_2_), δ 1.71–1.46 (2H, br, –SiCH_2_C*H*_2_CH_2_NH(CH_3_)_2_), δ 0.64 (2H, t, J = 8.47 Hz, –SiC*H*_2_CH_2_CH_2_NH(CH_3_)_2_). ^29^Si NMR (79.4 MHz, DMSO-*d*_6_): δ −67.0 (T^3^, T_8_-POSS).

### 3.5. Preparation of **Am-POSS(4)**

**Am-POSS(4)** was prepared similar to **Am-POSS(1)**, except that the starting material was TMTESPAC (purity: 98%, 0.612 g, 2.0 mmol). A white solid product (0.855 g) was obtained (99% yield based on the ideal chemical formula of one unit of this product*,* i.e.*,* [SiO_1.5_(CH_2_)_3_N(CH_3_)_3_ (CF_3_SO_2_)_2_N, FW = 433.5]). ^1^H NMR (400 MHz, DMSO-*d*_6_): δ 3.26–3.15 (2H, br, –SiCH_2_CH_2_C*H*_2_N(CH_3_)_3_), δ 3.03 (9H, s, –SiCH_2_CH_2_CH_2_N(C*H*_3_)_3_), δ 1.76–1.54 (2H, br, –SiCH_2_C*H*_2_CH_2_N(CH_3_)_3_), δ 0.64 (2H, t, J = 8.47 Hz, –SiC*H*_2_CH_2_CH_2_N(CH_3_)_3_). ^29^Si NMR (79.4 MHz, DMSO-*d*_6_): δ −67.3 (T^3^, T_8_-POSS).

### 3.6. Preparation of **Am-POSS(1,2)**

**Am-POSS(1,2)** was prepared similar to **Am-POSS(1)**, except that the starting material was a mixture of APTMS (purity: 96%, 0.187 g, 1.0 mmol) and MAPTMS (purity: 95%, 0.203 g, 1.0 mmol). A white solid product (0.761 g) was obtained (96% yield based on the ideal average chemical formula of one unit of this product, i.e., {[SiO_1.5_(CH_2_)_3_NH_3_ (CF_3_SO_2_)_2_N, FW = 391.4] × 0.50 + [SiO_1.5_(CH_2_)_3_NH_2_CH_3_ (CF_3_SO_2_)_2_N, FW = 405.4] × 0.50 = 398.4}). ^1^H NMR (400 MHz, DMSO-*d*_6_): δ 8.34–8.09 (2H, br, –SiCH_2_CH_2_CH_2_N*H*_2_CH_3_ of the MAPTMS component), δ 7.74–7.44 (3H, br, –SiCH_2_CH_2_CH_2_N*H*_3_ of the APTMS component), δ 2.89–2.78 (2H, br, –SiCH_2_CH_2_C*H*_2_NH_2_CH_3_ of the MAPTMS component), δ 2.78–2.68 (2H, br, –SiCH_2_CH_2_C*H*_2_NH_3_ of the APTMS component), δ 2.57 (3H, t, J = 5.27 Hz, –SiCH_2_CH_2_CH_2_NH_2_C*H*_3_ of the MAPTMS component), δ 1.68–1.40 (2H, br, –SiCH_2_C*H*_2_CH_2_NH_2_CH_3_ of the MAPTMS component and –SiCH_2_C*H*_2_CH_2_NH_3_ of the APTMS component), δ 0.76–0.47 (2H, br, –SiC*H*_2_CH_2_CH_2_NH_2_CH_3_ of the MAPTMS component and –SiC*H*_2_CH_2_CH_2_NH_3_ of the APTMS component). ^29^Si NMR (79.4 MHz, DMSO-*d*_6_): δ −66.6 and −66.8 (T^3^, T_8_-POSS).

### 3.7. Preparation of **Am-POSS(1,3)**

**Am-POSS(1,3)** was prepared similar to **Am-POSS(1)**, except that the starting material was a mixture of APTMS (purity: 96%, 0.187 g, 1.0 mmol) and DMAPTMS (purity: 96%, 0.216 g, 1.0 mmol). A brown and viscous solid product (0.804 g) was obtained (99% yield based on the ideal average chemical formula of one unit of this product, i.e., {[SiO_1.5_(CH_2_)_3_NH_3_ (CF_3_SO_2_)_2_N, FW = 391.4] × 0.50 + [SiO_1.5_(CH_2_)_3_NH(CH_3_)_2_ (CF_3_SO_2_)_2_N, FW = 419.4] × 0.50 = 405.4}). ^1^H NMR (400 MHz, DMSO-*d*_6_): δ 9.38–9.05 (1H, br, –SiCH_2_CH_2_CH_2_N*H*(CH_3_)_2_ of the DMAPTMS component), δ 7.79–7.40 (3H, br, –SiCH_2_CH_2_CH_2_N*H*_3_ of the APTMS component), δ 3.03–2.90 (2H, br, –SiCH_2_CH_2_C*H*_2_NH(CH_3_)_2_ of the DMAPTMS component), δ 2.83–2.65 (6H, br, –SiCH_2_CH_2_CH_2_NH(C*H*_3_)_2_ of the DMAPTMS component and 2H, br, –SiCH_2_CH_2_C*H*_2_NH_3_ of the APTMS component), δ 1.70–1.41 (2H, br, –SiCH_2_C*H*_2_CH_2_NH(CH_3_)_2_ of the DMAPTMS component and –SiCH_2_C*H*_2_CH_2_NH_3_ of the APTMS component), δ 0.77–0.42 (2H, br, –SiC*H*_2_CH_2_CH_2_NH(CH_3_)_2_ of the DMAPTMS component and –SiC*H*_2_CH_2_CH_2_NH_3_ of the APTMS component). ^29^Si NMR (79.4 MHz, DMSO-*d*_6_): δ −66.6 and −67.0 (T^3^, T_8_-POSS).

### 3.8. Preparation of **Am-POSS(1,4)**

**Am-POSS(1,4)** was prepared similar to **Am-POSS(1)**, except that the starting material was a mixture of APTMS (purity: 96%, 0.187 g, 1.0 mmol) and TMTESPAC (purity: 98%, 0.306 g, 1.0 mmol). A dark brown and viscous solid product (0.809 g) was obtained (98% yield based on the ideal average chemical formula of one unit of this product, i.e., {[SiO_1.5_(CH_2_)_3_NH_3_ (CF_3_SO_2_)_2_N, FW = 391.4] × 0.49 + [SiO_1.5_(CH_2_)_3_N(CH_3_)_3_ (CF_3_SO_2_)_2_N, FW = 433.5] × 0.51 = 412.9}). ^1^H NMR (400 MHz, DMSO-*d*_6_): δ 7.75–7.43 (3H, br, –SiCH_2_CH_2_CH_2_N*H*_3_ of the APTMS component), δ 3.25–3.13 (2H, br, –SiCH_2_CH_2_C*H*_2_N(CH_3_)_3_ of the TMTESPAC component), δ 3.08–2.92 (9H, br, –SiCH_2_CH_2_CH_2_N(C*H*_3_)_3_ of the TMTESPAC component), δ 2.81–2.68 (2H, br, –SiCH_2_CH_2_C*H*_2_NH_3_ of the APTMS component), δ 1.78–1.60 (2H, br, –SiCH_2_C*H*_2_CH_2_N(CH_3_)_3_ of the TMTESPAC component), δ 1.60–1.42 (2H, br, –SiCH_2_C*H*_2_CH_2_NH_3_ of the APTMS component), δ 0.78–0.44 (2H, br, –SiC*H*_2_CH_2_CH_2_N(CH_3_)_3_ of the TMTESPAC component and –SiC*H*_2_CH_2_CH_2_NH_3_ of the APTMS component). ^29^Si NMR (79.4 MHz, DMSO-*d*_6_): δ −66.6 and −67.3 (T^3^, T_8_-POSS).

### 3.9. Preparation of **Am-POSS(2,3)**

**Am-POSS(2,3)** was prepared similar to **Am-POSS(1)**, except that the starting material was a mixture of MAPTMS (purity: 95%, 0.203 g, 1.0 mmol) and DMAPTMS (purity: 96%, 0.216 g, 1.0 mmol). A brown and viscous product (0.779 g) was obtained (94% yield based on the ideal average chemical formula of one unit of this product, i.e., {[SiO_1.5_(CH_2_)_3_NH_2_CH_3_ (CF_3_SO_2_)_2_N, FW = 405.4] × 0.50 + [SiO_1.5_(CH_2_)_3_NH(CH_3_)_2_ (CF_3_SO_2_)_2_N, FW = 419.4] × 0.50 = 412.4}). ^1^H NMR (400 MHz, DMSO-*d*_6_): δ 9.34–9.09 (1H, br, –SiCH_2_CH_2_CH_2_N*H*(CH_3_)_2_ of the DMAPTMS component), δ 8.34–8.10 (2H, br, –SiCH_2_CH_2_CH_2_N*H*_2_CH_3_ of the MAPTMS component), δ 3.03–2.90 (2H, br, –SiCH_2_CH_2_C*H*_2_NH(CH_3_)_2_ of the DMAPTMS component), δ 2.88–2.79 (2H, br, –SiCH_2_CH_2_C*H*_2_NH_2_CH_3_ of the MAPTMS component), δ 2.79–2.65 (6H, br, –SiCH_2_CH_2_CH_2_NH(C*H*_3_)_2_ of the DMAPTMS component), δ 2.57 (3H, t, J = 5.04 Hz, –SiCH_2_CH_2_CH_2_NH_2_C*H*_3_ of the MAPTMS component), δ 1.71–1.39 (2H, br, –SiCH_2_C*H*_2_CH_2_NH(CH_3_)_2_ of the DMAPTMS component and –SiCH_2_C*H*_2_CH_2_NH_2_CH_3_ of the MAPTMS component), δ 0.75–0.43 (2H, br, –SiC*H*_2_CH_2_CH_2_NH(CH_3_)_2_ of the DMAPTMS component and –SiC*H*_2_CH_2_CH_2_NH_2_CH_3_ of the MAPTMS component). ^29^Si NMR (79.4 MHz, DMSO-*d*_6_): δ −67.0 and −67.1 (T^3^, T_8_-POSS).

### 3.10. Preparation of **Am-POSS(2,4)**

**Am-POSS(2,4)** was prepared similar to **Am-POSS(1)**, except that the starting material was a mixture of MAPTMS (purity: 95%, 0.203 g, 1.0 mmol) and TMTESPAC (purity: 98%, 0.306 g, 1.0 mmol). A dark brown and viscous product (0.828 g) was obtained (99% yield based on the ideal average chemical formula of one unit of this product, i.e., {[SiO_1.5_(CH_2_)_3_N H_2_CH_3_ (CF_3_SO_2_)_2_N, FW = 405.4] × 0.50 + [SiO_1.5_(CH_2_)_3_N(CH_3_)_3_ (CF_3_SO_2_)_2_N, FW = 433.5] × 0.50 = 419.5}). ^1^H NMR (400 MHz, DMSO-*d*_6_): δ 8.36–8.07 (2H, br, –SiCH_2_CH_2_CH_2_N*H*_2_CH_3_ of the MAPTMS component), δ 3.25–3.14 (2H, br, –SiCH_2_CH_2_C*H*_2_N(CH_3_)_3_ of the TMTESPAC component), δ 3.10–2.95 (9H, br, –SiCH_2_CH_2_CH_2_N(C*H*_3_)_3_ of the TMTESPAC component), δ 2.89–2.77 (2H, br, –SiCH_2_CH_2_C*H*_2_NH_2_CH_3_ of the MAPTMS component), δ 2.60–2.53 (3H, br, –SiCH_2_CH_2_CH_2_NH_2_C*H*_3_ of the MAPTMS component), δ 1.76–1.60 (2H, br, –SiCH_2_C*H*_2_CH_2_N(CH_3_)_3_ of the TMTESPAC component), δ 1.60–1.46 (2H, br, –SiCH_2_C*H*_2_CH_2_NH_2_CH_3_ of the MAPTMS component), δ 0.78–0.45 (2H, br, –SiC*H*_2_CH_2_CH_2_N(CH_3_)_3_ of the TMTESPAC component and –SiC*H*_2_CH_2_CH_2_NH_2_CH_3_ of the MAPTMS component). ^29^Si NMR (79.4 MHz, DMSO-*d*_6_): δ −66.8 and −67.3 (T^3^, T_8_-POSS).

### 3.11. Preparation of **Am-POSS(3,4)**

**Am-POSS(3,4)** was prepared similar to **Am-POSS(1)**, except that the starting material was a mixture of DMAPTMS (purity: 96%, 0.216 g, 1.0 mmol) and TMTESPAC (purity: 98%, 0.306 g, 1.0 mmol). A dark brown and viscous product (0.799 g) was obtained (94% yield based on the ideal average chemical formula of one unit of this product, i.e., {[SiO_1.5_(CH_2_)_3_N H(CH_3_)_2_ (CF_3_SO_2_)_2_N, FW = 419.4] × 0.50 + [SiO_1.5_(CH_2_)_3_N(CH_3_)_3_ (CF_3_SO_2_)_2_N, FW = 433.5] × 0.50 = 426.5}). ^1^H NMR (400 MHz, DMSO-*d*_6_): δ 9.35–9.12 (1H, br, –SiCH_2_CH_2_CH_2_N*H*(CH_3_)_2_ of the DMAPTMS component), δ 3.25–3.15 (2H, br, –SiCH_2_CH_2_C*H*_2_N(CH_3_)_3_ of the TMTESPAC component), δ 3.09–3.00 (9H, br, –SiCH_2_CH_2_CH_2_N(C*H*_3_)_3_ of the TMTESPAC component), δ 3.00–2.91 (2H, br, –SiCH_2_CH_2_C*H*_2_NH(CH_3_)_2_ of the DMAPTMS component), δ 2.81–2.68 (6H, br, –SiCH_2_CH_2_CH_2_NH(C*H*_3_)_2_ of the DMAPTMS component), δ 1.77–1.47 (2H, br, –SiCH_2_C*H*_2_CH_2_N(CH_3_)_3_ of the TMTESPAC component and –SiCH_2_C*H*_2_CH_2_NH(CH_3_)_2_ of the DMAPTMS component), δ 0.77–0.44 (2H, br, –SiC*H*_2_CH_2_CH_2_N(CH_3_)_3_ of the TMTESPAC component and –SiC*H*_2_CH_2_CH_2_NH(CH_3_)_2_ of the DMAPTMS component). ^29^Si NMR (79.4 MHz, DMSO-*d*_6_): δ −67.2 and −67.3 (T^3^, T_8_-POSS).

### 3.12. Measurements

The ^1^H and ^29^Si NMR spectra were recorded using an ECX-400 spectrometer (JEOL RESONANCE Inc., Tokyo, Japan). The absence of Cl and the elemental ratios of Si:S in the products were confirmed by EDX analyses using an XL30 scanning electron microscope (FEI Company, Hillsboro, OR, USA). The MALDI-TOF MS analyses of the products were performed using a Voyager Biospectrometry Workstation Ver. 5.1 (SHIMADZU Corporation, Kyoto, Japan) with 2,5-dihydroxybenzoic acid (DHB) as the matrix under positive ion mode. The DSC analyses were performed on a DSC-60 Plus (SHIMADZU Corporation, Kyoto, Japan). The samples placed in an aluminum capsule were cooled to −140 °C at a rate of 20 °C min^−1^ under a nitrogen flow (300 mL min^−1^) and then heated from −140 °C to 240 °C at the same rate. The *T*_g_ and *T*_m_ values were determined as the onset of the baseline shift and as the tops of the endothermic peaks, respectively, in the curves of the third scan (from −140 °C to 240 °C at a rate of 20 °C min^−1^) to eliminate the heat history in the samples. The XRD patterns were recorded at a scanning speed of 2*θ* = 9.0° min^−1^ using an X’Pert Pro diffractometer (PANalytical, Almelo, Netherlands) with Ni-filtered Cu Kα radiation (0.15418 nm). The TGA was performed on a TGA-50 (SHIMADZU Corporation, Kyoto, Japan) at a heating rate of 10 °C min^−1^ up to 1000 °C under nitrogen flow (100 mL min^−1^). The pyrolysis temperatures were determined from the 5% (*T*_d5_) and 10% (*T*_d10_) weight losses.

## 4. Conclusions

**Am-POSS(x,y)** were successfully prepared by the hydrolytic condensation in DMSO of mixtures of two starting materials, which were selected from the organotrialkoxysilanes containing primary, secondary, and tertiary amines and a quaternary ammonium salt, in the presence of HNTf_2_ and water. The products consisted of T_8_-POSS only. The DSC curves of **Am-POSS(1,3)**, **Am-POSS(1,4)**, and **Am-POSS(2,4)** did not show the endothermic peaks due to *T*_m_s, indicating their amorphous structures. Conversely, those of **Am-POSS(1,2)**, **Am-POSS(2,3)**, and **Am-POSS(3,4)** showed the endothermic peaks due to *T*_m_s, indicating their crystalline structures. XRD data also supported these results. These results indicate that the crystalline or amorphous structure of **Am-POSS(x,y)** can be controlled by the side-chain group combinations. **Am-POSS(1,3)**, **Am-POSS(1,4)**, and **Am-POSS(2,4)** were PILs with relatively low flow temperatures, while **Am-POSS(1,2)**, **Am-POSS(2,3)**, and **Am-POSS(3,4)** were PILs with relatively high flow temperatures.

## Figures and Tables

**Table 1:** The solubility of **Am-POSS(x)**.

Am-POSS(x)	Water	DMSO	DMF	Methanol	Acetone	Acetonitrile	Chloroform	Toluene	*n*-Hexane
**Am-POSS(1)**	+	+	+	+	+	+	−	−	−
**Am-POSS(2)**	−	+	+	+	+	+	−	−	−
**Am-POSS(3)**	−	+	+	+	+	+	−	−	−
**Am-POSS(4)**	−	+	+	+	+	+	−	−	−

+: Soluble at room temperature, −: Insoluble at room temperature; DMSO: Dimethyl sulfoxide, DMF: *N,N*-dimethylformamide.

**Table 2:** The solubility of **Am-POSS(x,y)**.

Am-POSS(x,y)	Water	DMSO	DMF	Methanol	Acetone	Acetonitrile	Chloroform	Toluene	*n*-Hexane
**Am-POSS(1,2)**	−	+	+	+	+	+	−	−	−
**Am-POSS(1,3)**	−	+	+	+	+	+	−	−	−
**Am-POSS(1,4)**	−	+	+	+	+	+	−	−	−
**Am-POSS(2,3)**	−	+	+	+	+	+	−	−	−
**Am-POSS(2,4)**	−	+	+	+	+	+	−	−	−
**Am-POSS(3,4)**	−	+	+	+	+	+	−	−	−

+: Soluble at room temperature, −: Insoluble at room temperature; DMSO: Dimethyl sulfoxide, DMF: *N,N*-dimethylformamide.

**Table 3:** Thermal properties of **Am-POSS(x,y)**.

Run	Am-POSS(x,y)	*T*_g_/°C ^(a)^	*T*_m_/°C ^(a)^	Flow Temp./°C ^(b)^	*T*_d5_/°C ^(c)^	*T*_d10_/°C ^(c)^
1	**Am-POSS(1,2)**	ND ^(d)^	154	140	384	397
2	**Am-POSS(1,3)**	−6	ND ^(d)^	45	374	395
3	**Am-POSS(1,4)**	15	ND ^(d)^	60	380	401
4	**Am-POSS(2,3)**	ND ^(d)^	104	95	371	387
5	**Am-POSS(2,4)**	1	ND ^(d)^	45	378	395
6	**Am-POSS(3,4)**	−2	86,118	95	383	400

(a) Determined as the onset of baseline shift in the curve of 3rd scan by DSC measurement. (b) Determined by visual observation. (c) Determined by TGA. (d) Not detected.

## Supplementary Materials

The following are available online at https://www.mdpi.com/1420-3049/24/24/4553/s1, Figure S1: EDX patterns of **Am-POSS(x)**., Figure S2: MALDI-TOF MS analysis of **Am-POSS(1)**., Figure S3: MALDI-TOF MS analysis of **Am-POSS(2)**., Figure S4: MALDI-TOF MS analysis of **Am-POSS(3)**., Figure S5: MALDI-TOF MS analysis of **Am-POSS(4)**., Figure S6: EDX patterns of **Am-POSS(x,y)**., Figure S7: MALDI-TOF MS analysis of **Am-POSS(1,2)**., Figure S8: MALDI-TOF MS analysis of **Am-POSS(1,3)**., Figure S9: MALDI-TOF MS analysis of **Am-POSS(1,4)**., Figure S10: MALDI-TOF MS analysis of **Am-POSS(2,3)**., Figure S11: MALDI-TOF MS analysis of **Am-POSS(2,4)**., Figure S12: MALDI-TOF MS analysis of **Am-POSS(3,4)**., Figure S13: TGA thermograms of **Am-POSS(x,y)**., Figure S14: Photograph of the apparatus.
