# Supplementary material for: Control of Crystalline-Amorphous Structures of Polyhedral Oligomeric Silsesquioxanes Containing Two Types of Ammonium Side-Chain Groups and Their Properties as Protic Ionic Liquids
Source: Molecules. 2019 Dec 12;24(24):4553. doi: 10.3390/molecules24244553 (PMC6943538; doi:10.3390/molecules24244553)
Supplement: Supplementary file 1 [file molecules-24-04553-s001.pdf]

Supplementary Material

Control of crystalline-amorphous structures of polyhedral oligomeric silsesquioxanes containing two types of ammonium side-chain groups and their properties as protic ionic liquids

Ryoya Hasebe[1] and Yoshiro Kaneko[1,*]

[1]Graduate School of Science and Engineering, Kagoshima University, 1-21-40 Korimoto, Kagoshima 890-0065
*Correspondence: ykaneko@eng.kagoshima-u.ac.jp

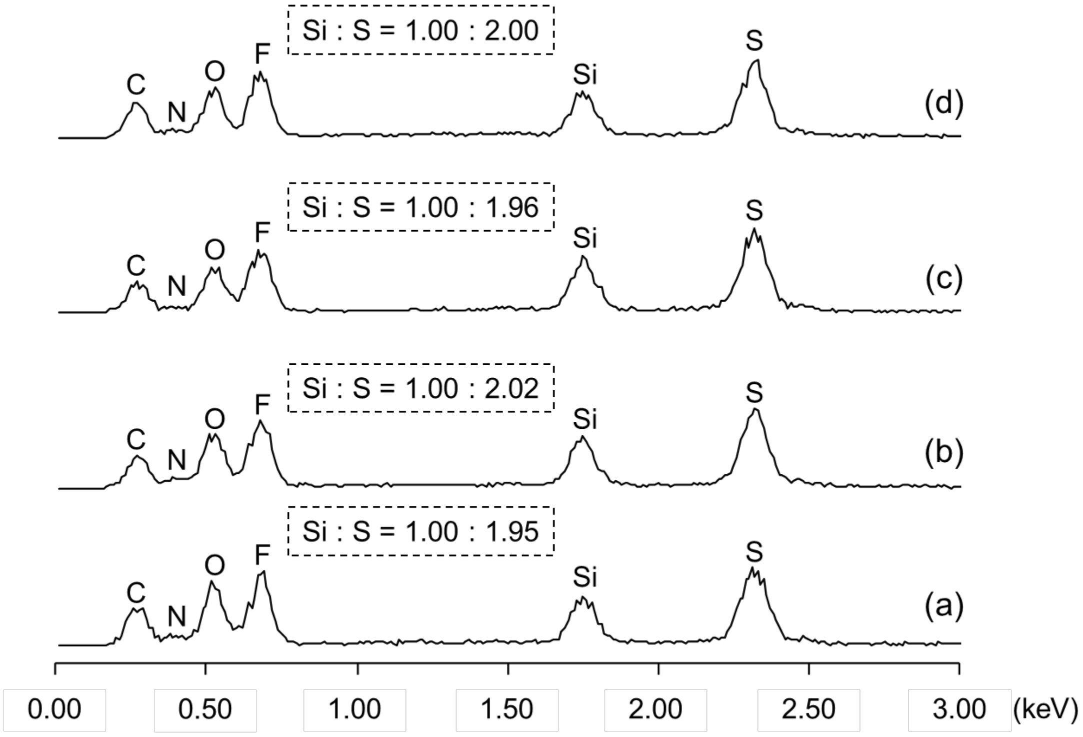

Figure S1. EDX patterns of (a) **Am-POSS(1)**, (b) **Am-POSS(2)**, (c) **Am-POSS(3)**, and (d) **Am-POSS(4)**.

	Number of side-chain group (R')			Calcd m/z	Found m/z
	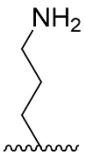	$H^{\oplus}$	$(CF_3SO_2)_2N^{\ominus}$		
(i)	8	1	0	881.3	881.4

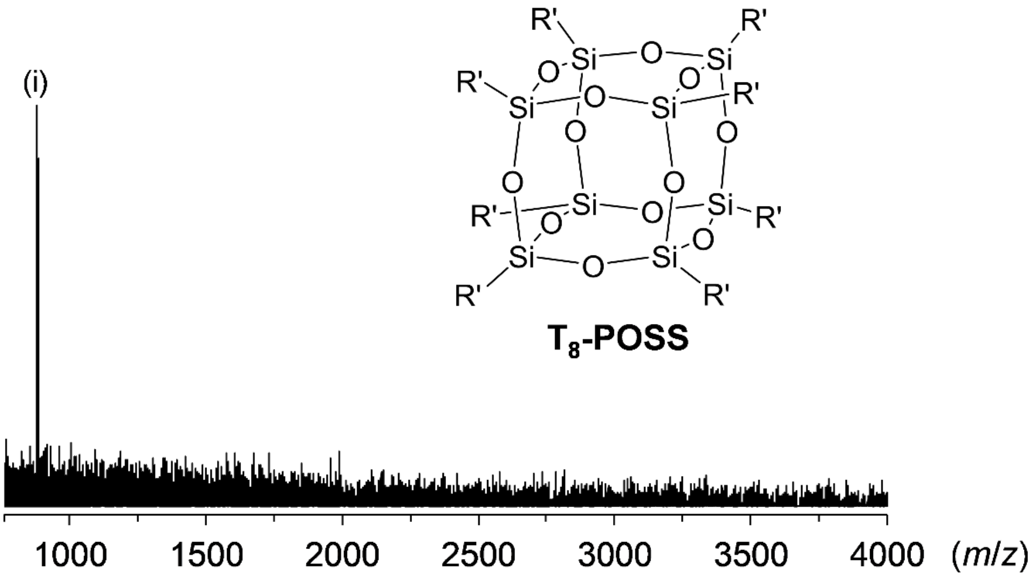

Figure S2. MALDI-TOF MS analysis of **Am-POSS(1)**.

	Number of side-chain group (R')			Calcd m/z	Found m/z
	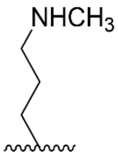	$H^{\oplus}$	$(CF_3SO_2)_2N^{\ominus}$		
(i)	8	1	0	993.4	993.5

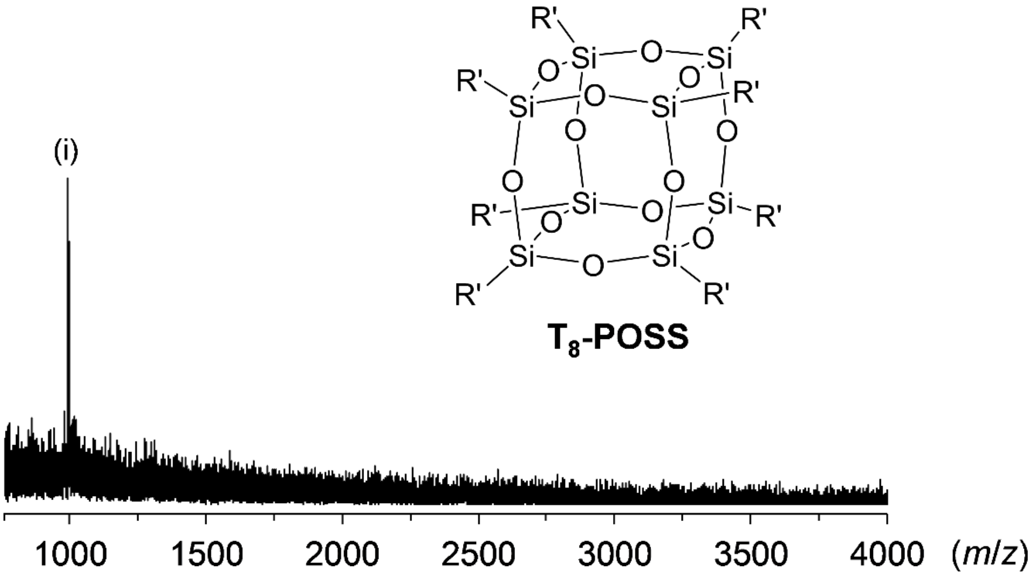

Figure S3. MALDI-TOF MS analysis of **Am-POSS(2)**.

	Number of side-chain group (R')			Calcd *m/z*	Found *m/z*
	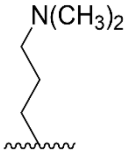	$H^{\oplus}$	$(CF_3SO_2)_2N^{\ominus}$		
(i)	8	1	0	1105.5	1105.9

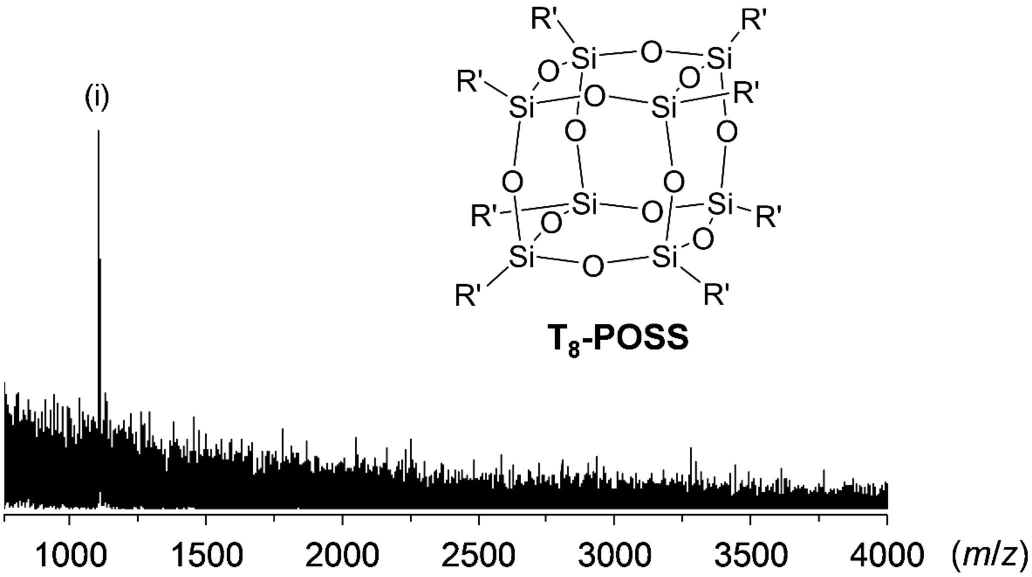

Figure S4. MALDI-TOF MS analysis of **Am-POSS(3)**.

	Number of side-chain group (R')			Calcd *m*/*z*	Found *m*/*z*
	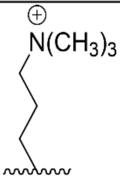 $\overset{\oplus}{N}(CH_3)_3$	$H^{\oplus}$	$(CF_3SO_2)_2N^{\ominus}$		
(i)	8	0	7	3184.2	3184.2

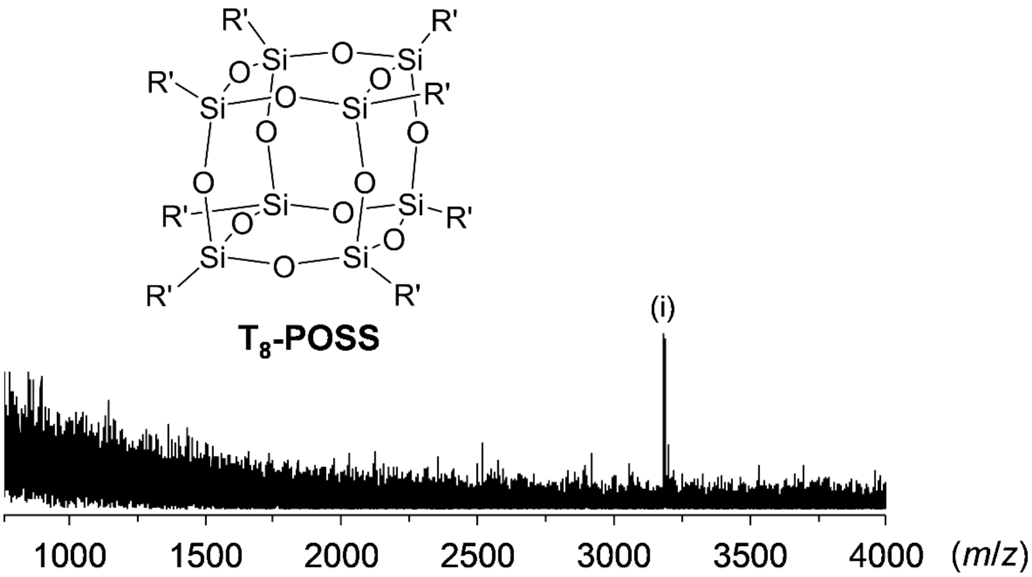

Figure S5. MALDI-TOF MS analysis of **Am-POSS(4)**.

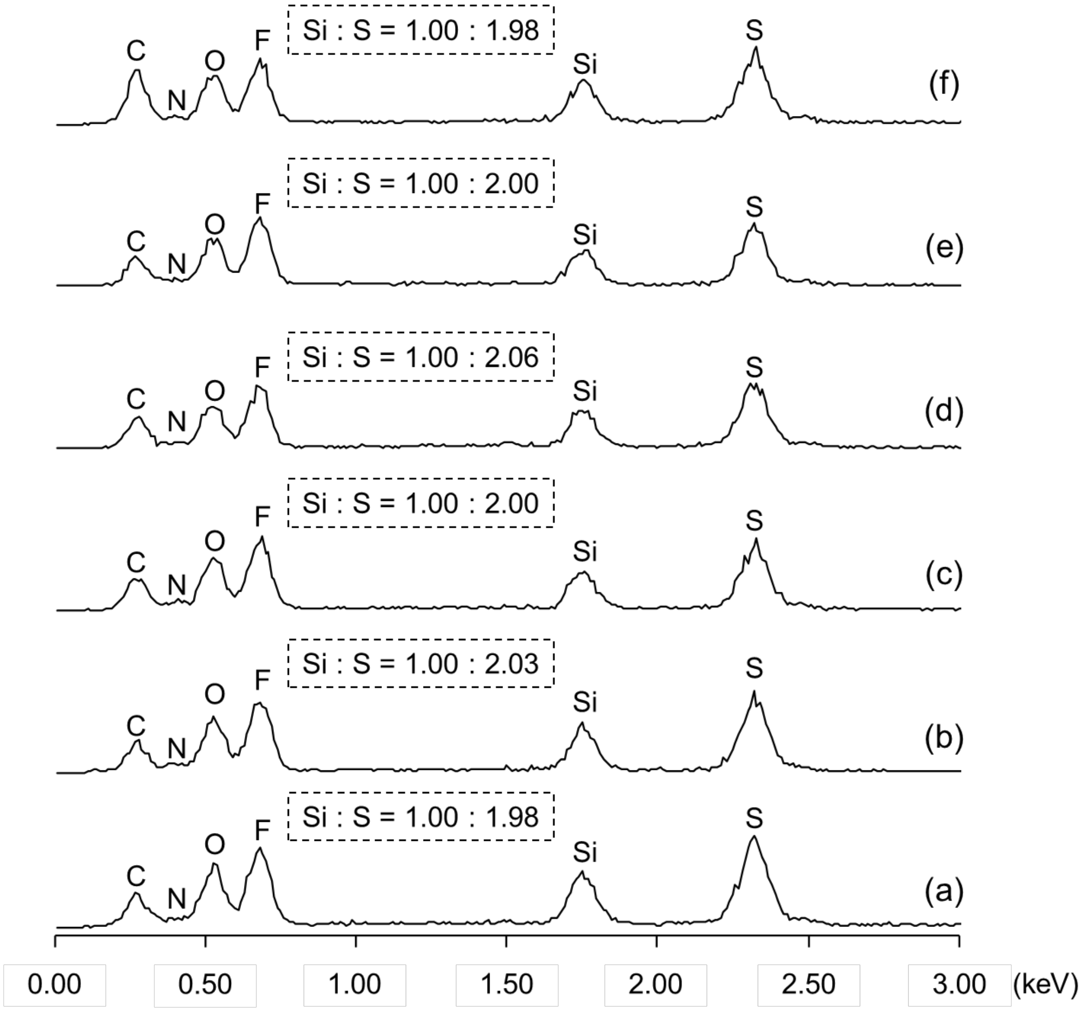

Figure S6. EDX patterns of (a) **Am-POSS(1,2)**, (b) **Am-POSS(1,3)**, (c) **Am-POSS(1,4)**, (d) **Am-POSS(2,3)**, (e) **Am-POSS(2,4)**, and (f) **Am-POSS(3,4)**.

	Number of side-chain group (R')				Calcd *m*/*z*	Found *m*/*z*
	NH_2	$NHCH_3$	$H^{\oplus}$	$(CF_3SO_2)_2N^{\ominus}$		
(i)	1	7	1	0	979.4	979.7
(ii)	2	6	1	0	965.4	965.6
(iii)	3	5	1	0	951.4	951.6
(iv)	4	4	1	0	937.4	937.6
(v)	5	3	1	0	923.3	923.6
(vi)	6	2	1	0	909.3	909.6
(vii)	7	1	1	0	895.3	895.5

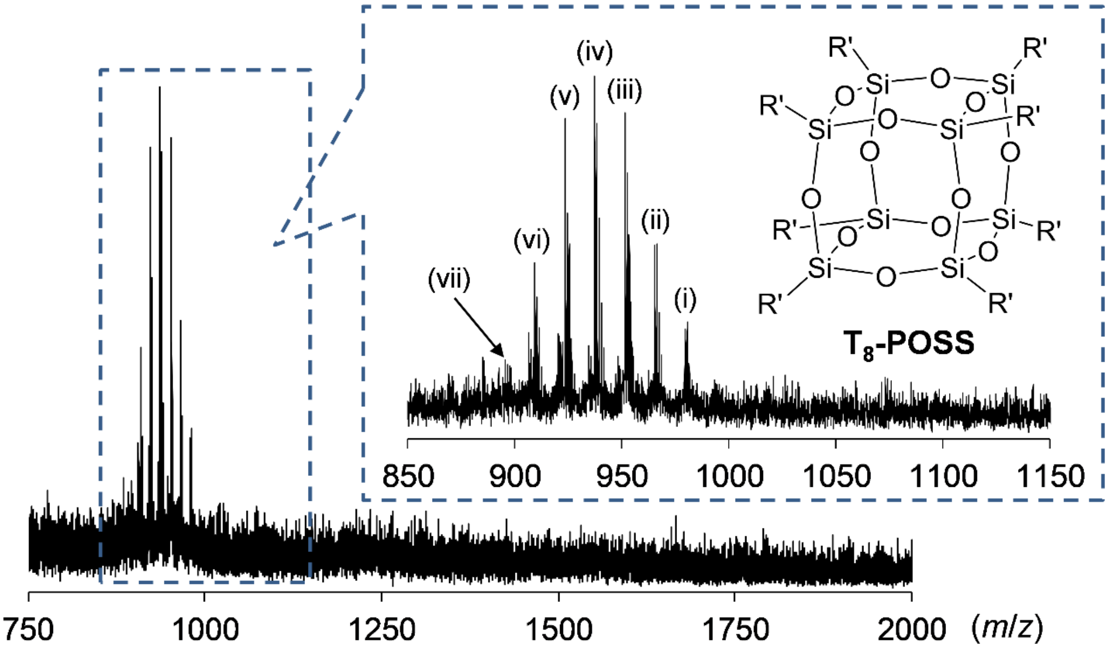

Figure S7. MALDI-TOF MS analysis of **Am-POSS(1,2)**.

	Number of side-chain group (R')				Calcd *m*/*z*	Found *m*/*z*
	$NHCH_3$	$N(CH_3)_2$	$H^{\oplus}$	$(CF_3SO_2)_2N^{\ominus}$		
(i)	1	7	1	0	1091.5	1091.7
(ii)	2	6	1	0	1077.5	1077.7
(iii)	3	5	1	0	1063.5	1063.7
(iv)	4	4	1	0	1049.5	1049.8
(v)	5	3	1	0	1035.5	1035.7
(vi)	6	2	1	0	1021.4	1021.7
(vii)	7	1	1	0	1007.4	1007.8

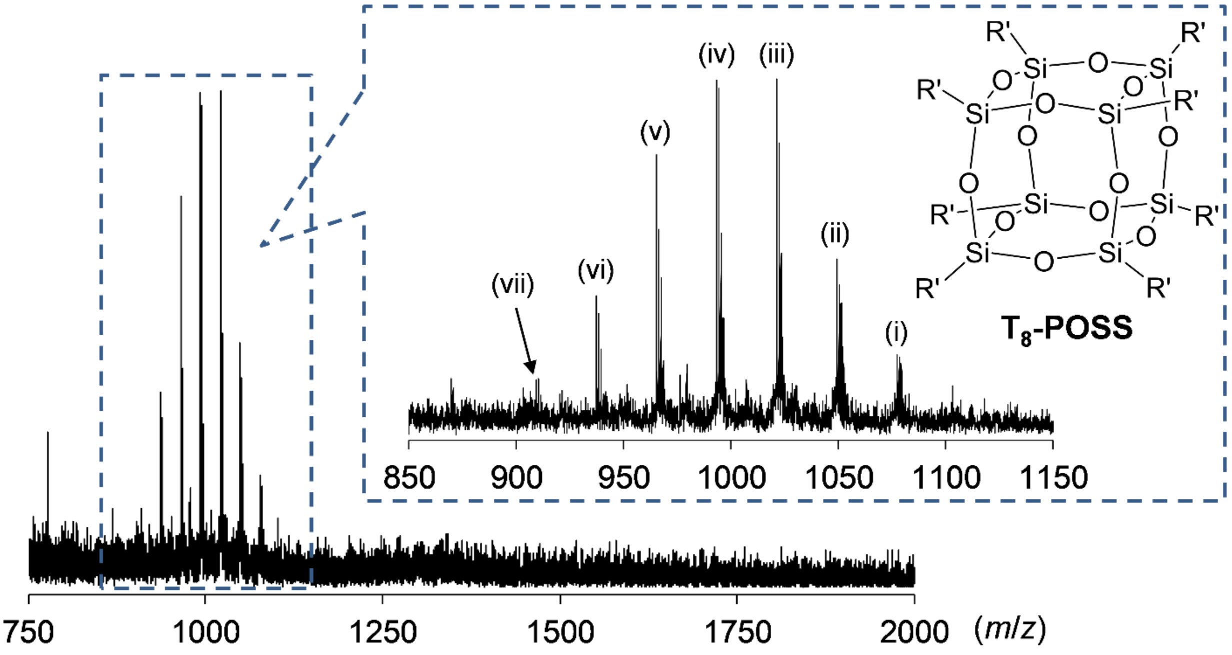

Figure S8. MALDI-TOF MS analysis of **Am-POSS(1,3)**.

	Number of side-chain group (R')				Calcd *m*/*z*	Found *m*/*z*
	NH_2	$N^{\oplus}(CH_3)_3$	$H^{\oplus}$	$(CF_3SO_2)_2N^{\ominus}$		
(i)	1	7	0	6	2861.2	2861.6
(ii)	2	6	0	5	2538.2	2537.8
(iii)	3	5	0	4	2215.2	2215.0
(iv)	4	4	0	3	1892.3	1892.2
(v)	5	3	0	2	1569.3	1569.4
(vi)	6	2	0	1	1246.3	1246.5
(vii)	7	1	0	0	923.3	923.6

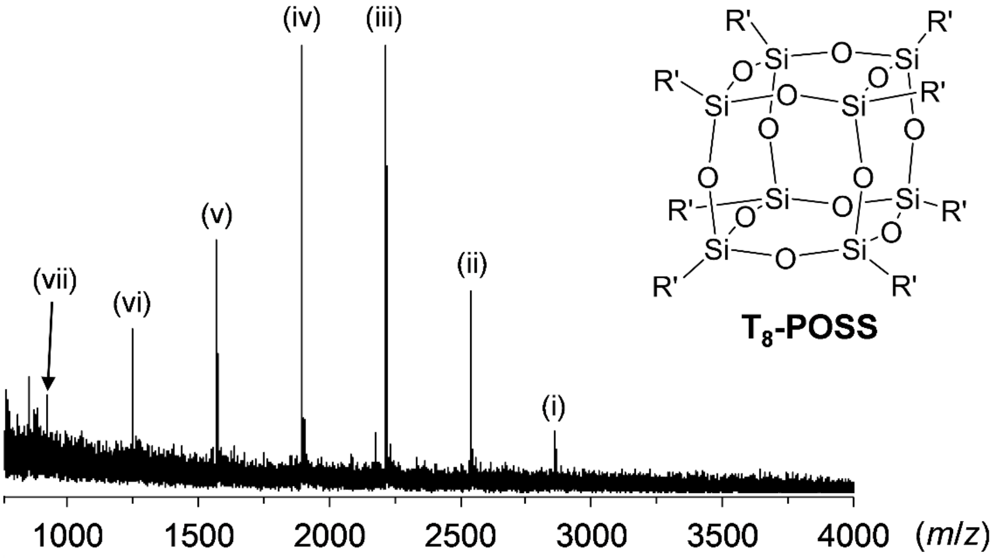

Figure S9. MALDI-TOF MS analysis of **Am-POSS(1,4)**.

	Number of side-chain group (R')				Calcd m/z	Found m/z
	$NHCH_3$	$N(CH_3)_2$	$H^{\oplus}$	$(CF_3SO_2)_2N^{\ominus}$		
(i)	1	7	1	0	1091.5	1091.7
(ii)	2	6	1	0	1077.5	1077.7
(iii)	3	5	1	0	1063.5	1063.7
(iv)	4	4	1	0	1049.5	1049.8
(v)	5	3	1	0	1035.5	1035.7
(vi)	6	2	1	0	1021.4	1021.7
(vii)	7	1	1	0	1007.4	1007.8

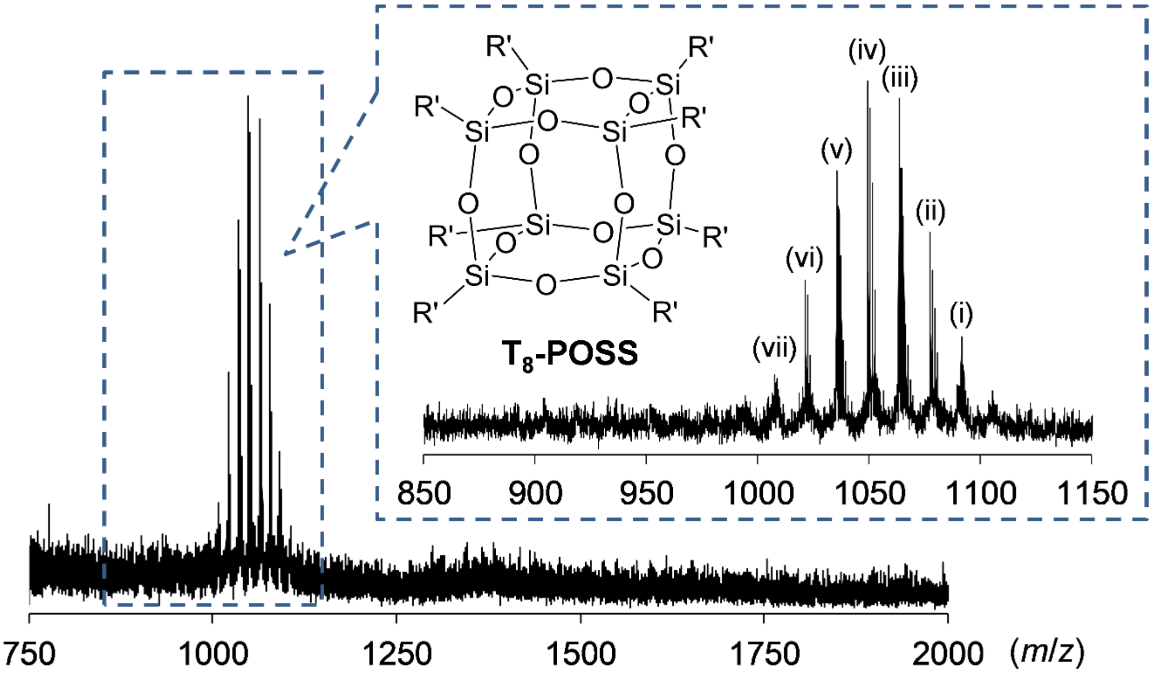

Figure S10. MALDI-TOF MS analysis of **Am-POSS(2,3)**.

	Number of side-chain group (R')				Calcd m/z	Found m/z
	$NHCH_3$	$N^{\oplus}(CH_3)_3$	$H^{\oplus}$	$(CF_3SO_2)_2N^{\ominus}$		
(i)	2	6	0	5	2566.2	2566.6
(ii)	3	5	0	4	2257.3	2257.8
(iii)	4	4	0	3	1948.3	1948.0
(iv)	5	3	0	2	1639.4	1639.3
(v)	6	2	0	1	1330.4	1330.4
(vi)	7	1	0	0	1021.4	1021.7

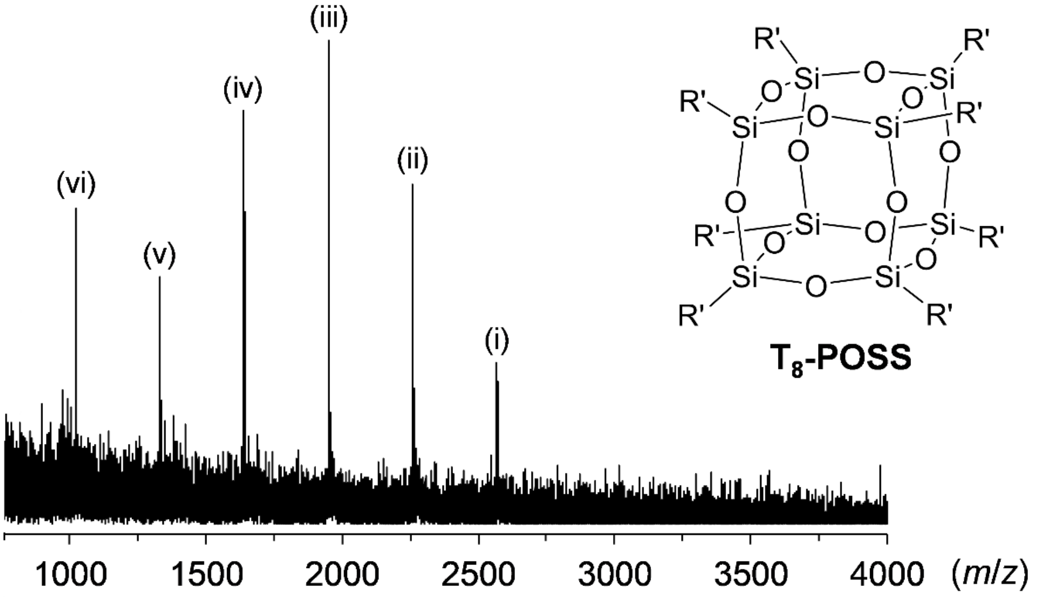

Figure S11. MALDI-TOF MS analysis of **Am-POSS(2,4)**.

	Number of side-chain group (R')				Calcd *m/z*	Found *m/z*
	$N(CH_3)_2$	$\overset{\oplus}{N}(CH_3)_3$	$H^{\oplus}$	$(CF_3SO_2)_2N^{\ominus}$		
(i)	1	7	0	6	2889.2	2889.0
(ii)	2	6	0	5	2594.3	2594.3
(iii)	3	5	0	4	2299.3	2299.6
(iv)	4	4	0	3	2004.4	2004.9
(v)	5	3	0	2	1709.4	1709.2
(vi)	6	2	0	1	1414.5	1414.5
(vii)	7	1	0	0	1119.6	1119.6

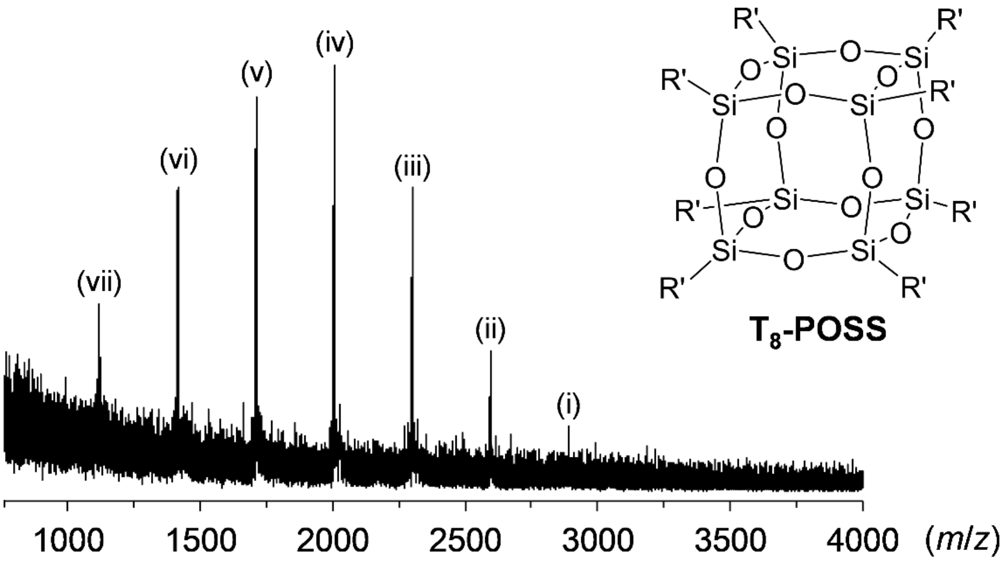

T_8-POSS

Figure S12. MALDI-TOF MS analysis of **Am-POSS(3,4)**.

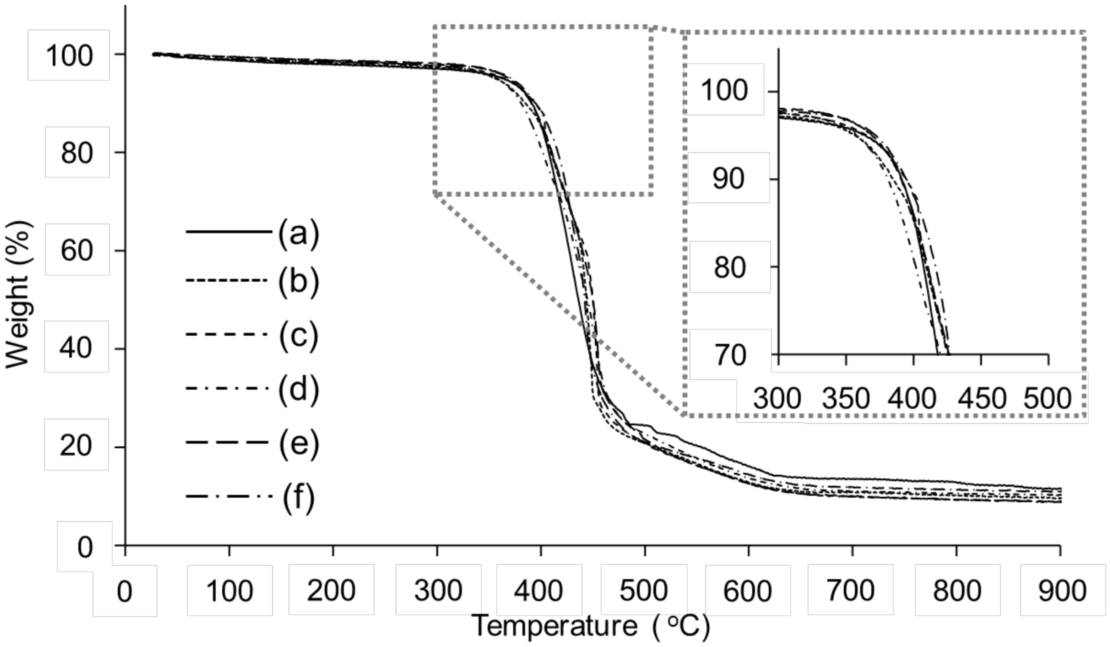

Figure S13. TGA thermograms of (a) **Am-POSS(1,2)**, (b) **Am-POSS(1,3)**, (c) **Am-POSS(1,4)**, (d) **Am-POSS(2,3)**, (e) **Am-POSS(2,4)**, and (f) **Am-POSS(3,4)** under nitrogen flow.

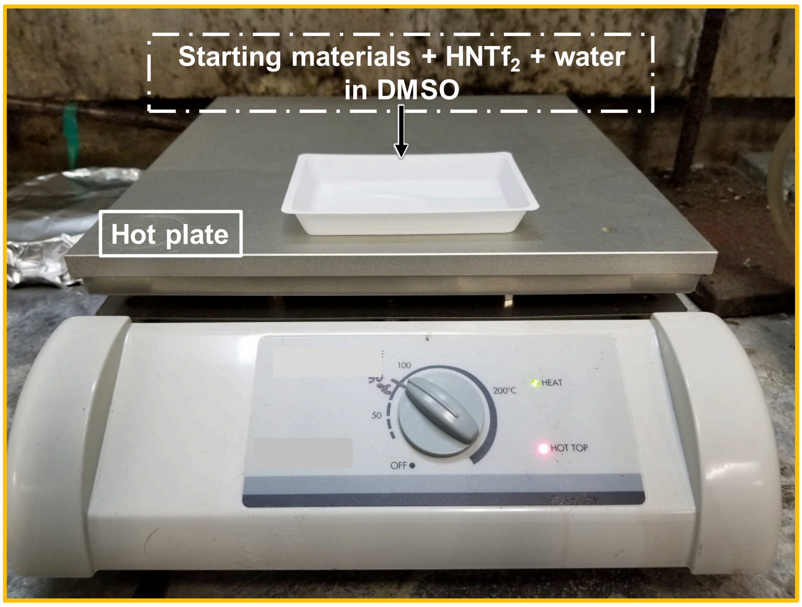

Figure S14. Photograph of the apparatus (hot plate), where the reactions were performed at *ca.* 60 °C.
